# Catabolism of diethyl ether as a sole carbon and energy source by *Mycolicibacterium* sp. ELW1

**DOI:** 10.1007/s10532-026-10378-8

**Published:** 2026-10-06

**Authors:** John B. Joyce, Eric S. Miller, Michael R. Hyman

**Affiliations:** Department of Plant and Microbial Biology, North Carolina State University, Raleigh, NC 27695-7615 USA

**Keywords:** Diethyl ether, Megaplasmid, Isobutylene, Monooxygenase, Dehydrogenases, Transcriptomics

## Abstract

The model isobutylene (IB)-metabolizing strain *Mycolicibacterium* sp. ELW1 grew aerobically on diethyl ether (DEE) as a sole carbon and energy source. DEE supported an equivalent biomass yield to IB despite strong effects of DEE concentration on growth rate and lag phase. Differential expression analysis of the transcriptome revealed extensive transcriptional remodeling during growth on DEE compared to fructose, including the substantial upregulation of chromosomally-encoded alcohol and acetaldehyde dehydrogenases, and the plasmid‑encoded isobutylene monooxygenase (IMO). In resting‑cell assays, IB‑grown cells oxidized DEE twice as rapidly as DEE‑grown cells and ethanol and acetaldehyde temporarily accumulated during DEE oxidation. In contrast, no products were detected during DEE oxidation by DEE-grown cells. Loss of the megaplasmid pELW1‑1 prevented growth on both IB and DEE while activity‑based labeling (ABL) analyses suggest the plasmid-encoded IMO is the major diyne‑reactive monooxygenase in both IB and DEE-grown cells. Supplementing cultures growing on DEE with small amounts of IB increased both DEE consumption rates and biomass yields, and this effect appears to involve decreases in the lag phase associated with growth on DEE alone. In contrast to some previous studies of DEE catabolism which have concluded DEE is unable to induce expression of soluble di-iron monooxygenases (SDIMOs), collectively, our results indicate DEE catabolism leads to substantial expression of the plasmid‑encoded IMO and is supported by expression of chromosomally encoded alcohol and aldehyde dehydrogenases.

**Supplementary Information:**

The online version contains supplementary material available at https://doi.org/10.1007/s10532-026-10378-8.

## Introduction

The ether bond is an important structural feature of many anthropogenic compounds that are generally resistant to biodegradation (White et al. 1996). Prominent examples include the gasoline oxygenate methyl *tertiary* butyl ether (MTBE) and the chlorinated solvent stabilizer, 1, 4-dioxane (14D). Diethyl ether (DEE) is another example, and this compound is widely used in the industrial production of plastics, paints, coatings, perfumes, and other cosmetics. This simple alkyl ether is also a solvent for diverse organics including fats, waxes, oils, resins, and dyes and it has also been used as an anesthetic, an alcohol denaturant, a fuel oxygenate, and as part of cold weather starting fluids for combustion engines (Monticelli 2014).

Diverse microorganisms can grow aerobically on DEE as a sole source of carbon and energy. The hydrocarbon-oxidizing fungus, *Graphium* sp. initiates DEE catabolism through the O-dealkylating activity of a hydrocarbon-inducible cytochrome P450 (Hardison et al. 1997). Among bacteria, DEE-oxidizing strains were first detected in soils (Heydeman 1974) while more recently multiple DEE-utilizing strains were isolated from sewage sludge (Kim and Engesser 2004). A prominent strain among these isolates (*Rhodococcus* sp. DEE5151) grows on a wide range of symmetrical *n*-alkyl ethers (C_2_–C_7_) (Kim and Engesser 2004). Like *Graphium* sp., DEE5151 is thought to O-dealkylate DEE through the activity of monooxygenases (Kim and Engesser 2004). *Pseudonocardia tetrahydrofuranoxidans* K1 is a well characterized tetrahydrofuran-(THF)-metabolizing strain that also grows on DEE (Kohlweyer et al. 2000). Another THF- and 14D-metabolizing *Pseudonocardia* strain, *P. dioxanivorans* CB1190, can also grow on DEE, butyl methyl ether, and a range of cyclic ethers (Parales et al. 1994). Like strain K1, the cyclic ether-oxidizing activity of strain CB1190 is catalyzed by tetrahydrofuran monooxygenase, a Group 5 soluble di-iron monooxygenase (SDIMO) (Sales et al. 2013). It seems likely, although unproven, that this enzyme is also responsible for initiating DEE catabolism in other *Pseudonocardia* strains.

In contrast, several aerobic strains have been identified that can oxidize DEE but, for a variety of reasons, are unable to use this ether as a sole source of carbon and energy for growth. The aerobic nitrifying bacterium *Nitrosomonas europaea* oxidizes DEE to ethanol and acetaldehyde through the activity of ammonia monooxygenase (Yilmaz et al. 1996). This autotrophic bacterium catabolizes few organic compounds and lacks the enzymes needed to further oxidize the metabolites generated during DEE oxidation. A similar situation exists with aerobic methanotrophs. The purified soluble methane monooxygenase (sMMO) from *Methylococcus capsulatus* (Bath) can oxidize DEE and generates equimolar amounts of ethanol and acetaldehyde during this reaction (Colby et al. 1977). Although some methanotrophs can slowly further oxidize ethanol through the activity of methanol dehydrogenase (Anthony and Zatman 1965), growth of methanotrophs on DEE has not been reported.

In other DEE-oxidizing organisms there appears to be a regulatory effect on monooxygenase expression that dictates the ability to utilize DEE as a growth-supporting substrate. For example, the fuel oxygenate-degrading strain *Aquincola tertiaricarbonis* L108 can oxidize DEE, MTBE, and other ether fuel oxygenates through the activity of a constitutively expressed cytochrome P450 (EthABCD) (Schäfer et al. 2011). In other ether-degrading strains expression of this enzyme is inducible and regulated by EthR which is absent from the *eth* operon in strain L108 (Schuster et al. 2013). In another example, *Burkholderia cepacia (vietnamiensis)* G4 expresses toluene 2-monooxygenase (T2MO), a Group 2 SDIMO, during growth on toluene. Both toluene-grown cells of strain G4 and purified T2MO oxidize DEE (Hur et al. 1997). Purified T2MO oxidizes DEE at an equivalent rate to toluene and generates equimolar amounts of ethanol and acetaldehyde as products. Despite this ability, in short term assays (≤ 36 h) strain G4 does not grow on DEE as a sole carbon and energy source. However, a mutant strain (G4/PR1) that constitutively expresses T2MO grows almost as rapidly on DEE (doubling time (T_d_) = 6 h) as toluene (T_d_ = 4 h). This study concluded that the differences between growth and non-growth on DEE “is dependent solely on the genetic regulation of toluene 2-monooxygenase” (Hur et al. 1997).

In this study we have characterized the oxidation of DEE and the DEE-dependent growth of the IB-metabolizing strain *Mycolicibacterium* sp. ELW1. This strain is one of the few bacteria known to grow aerobically on IB, and the catabolic pathway in all known IB-metabolizing strains involves the initial oxidation of IB to an epoxide (2-methyl-1,2-epoxypropane [IBO]) by another Group 2 SDIMO, isobutylene monooxygenase (IMO) (Kottegoda et al. 2015; Helbich et al. 2023; Joyce et al. 2026). As expected, this enzyme is structurally and catalytically very similar to other Group 2 SDIMOs used to initiate alkene oxidation by propylene-grown *Xanthobacter autotrophicus* Py2 and isoprene-grown *Rhodococcus* sp. strain AD45. An unusual feature of strain ELW1 is that unlike most other well characterized aerobic alkene-oxidizing bacteria, this bacterium transforms the initial monooxygenase-generated epoxide, IBO, into the diol 2-methyl-1,2-propanediol (MPD), using an epoxide hydrolase (IbcK) (Faulkner et al. 2025) rather than conjugating epoxides with either glutathione (van Hylckama Vlieg et al. 1999, 2000) or coenzyme M (CoM) (Allen et al. 1999; Coleman and Spain 2003). Further oxidation of MPD is putatively catalyzed sequentially by an alcohol dehydrogenase (IbcH) and an aldehyde dehydrogenase (IbcJ) to 2-hydroxyisobutyric acid (2-HIBA). The genes encoding the enzymes that initially oxidize IB to 2-HIBA are all encoded and co-located in a catabolic island on a 221 kbp megaplasmid, pELW1-1 (Joyce et al. 2026). The genes encoding the enzymes required for the further oxidation of 2-HIBA are also co-located in a separate island on this plasmid.

The overall aim of this study was to investigate the abilities of strain ELW1 to oxidize DEE and to use this compound as a sole source of carbon and energy. Emphasis has been placed on investigating not only the role of monooxygenases but also the less well studied alcohol and aldehyde dehydrogenases thought to be involved in these processes. The results of this study suggest DEE catabolism by strain ELW1 is initiated primarily by the plasmid-encoded monooxygenase IMO but also involves the induction and activities of chromosomally encoded dehydrogenases.

## Materials and methods

### Reagents

The chemicals used in this study, their purities, and the suppliers were as follows. Alexa Fluor™ 647 Azide, triethylammonium salt (99% purity), Invitrogen (Grand Island, NY); Tris-hydroxypropyltriazolylmethylamine (THPTA; 95% purity), Lumiprobe (Hunt Valley, MD); 1,5-hexadiyne (15HD; 50%, vol/vol, in *n*-pentane), 1,2-epoxy-2 methylpropane (isobutylene oxide [IBO]) (> 99% purity), and isoprene (99% purity) was obtained from Alfa Aesar (Ward Hill, MA); 1,6-heptadiyne (16HD) (97% purity), 1,7-octadiyne (17OD) (98% purity), 1,4-diethynylbenzene (DEB) (96% purity), dipropargylamine (DIP) (97% purity), acetaldehyde (99% purity), 1-butene (> 99% purity), 2-butene; racemic mixture of *cis* & *trans* forms (> 99% purity), *cis*−2-butene (> 99% purity), *trans*−2-butene (> 99% purity), ethylene (> 99.5% purity), fructose (> 99% purity), 2-hydroxyisobutyric acid (2-HIBA) (99% purity), 2-methyl-2-butene (> 99% purity), 3-methyl-1-butene (95% purity), 2-methylpropene (isobutylene [IB]) (99% purity), propene (> 99% purity), methane (> 99% purity), propyne (98% purity), 1-octyne (97% purity), and dimethyl sulfoxide (DMSO) (99.5% purity) were obtained from Sigma-Aldrich Chemical Co. (Milwaukee, WI); 1,8-nonadiyne (18ND) (> 95% purity) and 1,9-decadiyne (19DD) (> 98% purity) were obtained from Tokyo Chemical Industry (Tokyo, Japan); Lysing Matrix B, Bulk 0.1 mm silica spheres, MP Biomedicals™ (Santa Ana, CA); PageRuler Prestained NIR Protein Ladder and diethyl ether (99+%, pure, stabilized with BHT) were obtained from Thermo Scientific (Waltham, MA); absolute ethanol was obtained from Aaper Alcohol and Chemical Co. (Shelbyville, KY); 2-methyl-1,2-propanediol (MPD) was obtained from Oakwood Chemical Co. (Estill, SC); 2-methylpropane (isobutane) (CP grade), and compressed gases (H_2_, N_2_, and air) used for gas chromatography were obtained from local industrial vendors (Airgas, Raleigh, NC).

### Bacterial cultivation and media

*Mycolicibacterium* sp. strain ELW1 was maintained on mineral salt medium (MSM) agar plates in a glass desiccator containing isobutylene (IB) (∼10%, v/v, gas phase) and passaged every 3–4 weeks for the duration of this study (Kottegoda et al. 2015). *Mycolicibacterium* sp. strain ELW1ΔpELW1-1 was maintained on plate count agar (PCA) that contained casein-yeast extract-dextrose (CYD) medium (Becton, Dickinson and Co., Sparks, MD) and was passaged every 3–4 weeks for the duration of this study.

Cultures of ELW1 were routinely grown on IB in 25 or 100 mL of MSM in previously autoclaved 160 or 700 mL glass serum bottles, respectively (Wheaton Scientific, Millville, NJ). All bottles were fitted with sterile butyl rubber septa and sealed with aluminum crimp caps (160 mL bottles) or sterile open-top caps fitted with butyl rubber septa (700 mL bottles). Unless otherwise stated, all cultures were inoculated to an initial OD_600_ ≤0.01–0.05 with a suspension of cells previously grown on MSM and IB (10%, v/v, gas phase). After inoculation, IB (~ 10%, v/v, gas phase) was added as an overpressure to the sealed bottle using sterile plastic syringes fitted with a sterile disposable 0.1 μm filter (Millipore Co., Bedford, MA). The bottles were incubated at 30 ℃ in the dark using a shaker operated at 150 rpm. Growth was measured as optical density at 600 nm (OD_600_) with a spectrophotometer (WPA CO8000 Cell Density Meter; Biochrom, Cambridge, U.K.) (1.0 OD_600_ =~0.25 mg/mL total protein). To eliminate any effect of flocculation on OD measurements, cultures were vortexed for 1 min immediately prior to optical density measurements. To confirm the purity of cultures after growth, a culture sample (25 µL) was streaked onto non-selective plate count agar (PCA) plates.

Cells were typically harvested by centrifugation (10,000 RCF; 5 min; 4 ℃), washed three times in phosphate buffer (PB; 50 mM NaH_2_PO_4_, pH 7.0) (15 mL), and then finally resuspended in PB (1 mL). Culture protein concentrations were routinely determined by resuspended cell pellets in PB (250 µL). Lysis matrix B beads (~0.075–0.15 g) were then added to the cell suspension and the cells were broken using three sequential cycles of 120 s at 5 m/s using a Bead Mill 4 (Fisher Brand). The resulting lysate was then centrifuged (17,000 RCF; 10 min; 4 ℃) to remove unbroken cells and cellular debris. The resulting supernatant was retained, and the protein concentration was determined using the BCA protein assay.

Batch culture growth was measured on fructose, DEE, IBO, MPD, 2-HIBA, acetaldehyde, ethanol, 1-butene, 2-butene, *cis-*2-butene, *trans-*2-butene, 2-methyl-2-butene, 3-methyl-1-butene, methane, propane, 2-methylpropane, ethylene, propene, isoprene, propyne, or a combination of these substrates, as described above. Fructose, IBO, MPD, and 2-HIBA were added as filter-sterilized aqueous stock solutions (1 M). DEE, acetaldehyde, and ethanol were added as neat compounds. All gases were added to the headspace as over-pressures. All carbon sources were added at the volumes or amounts specified below for specific experiments. For the plasmid-free strain ELW1ΔpELW1-1, growth was monitored for up to 28 days.

### Gas chromatography

Reactant and product concentrations in all experiments were quantified by gas chromatography (GC), as described previously (Kottegoda et al. 2015) with the following modifications. For gaseous-phase analytes, samples (10 μL) were taken directly from the headspace of culture bottles using gas-tight syringes and were immediately injected into a Shimadzu GC-14A gas chromatograph (Kyoto, Japan) fitted with a flame ionization detector and a RT-Alumina BOND/Na_2_SO_4_ capillary column (30 m by 10 µm [inside diameter{i.d.}], 0.53 mm; Restek (Bellefonte, PA). The injection and detector temperatures were 220 ℃ and 200 ℃, respectively, and N_2_ was used as the carrier gas at a flow rate of 15 mL min^−1^. A column temperature of 35 ℃ was used for the analysis of IB and DEE.

To quantify the aqueous-phase analytes, samples (2 μL) were analyzed using Shimadzu GC-8A gas chromatograph or GC-14A gas chromatographs fitted with flame ionization detectors and stainless-steel columns (0.3 by 183 cm) or (0.3 by 61 cm), respectively, filled with Porapak Q (80/100 mesh; Waters Associates, Framingham, MA). The GC-8A was operated with an injector and detector port temperature of 220 ℃. Nitrogen was used as the carrier gas at a flow rate of 15 mL min^−1^. A column temperature of 155 ℃ was used for the analysis of acetaldehyde, ethanol, and DEE. A column temperature of 180 ℃ was used for the detection of *tertiary*-butyl alcohol (TBA). MPD was analyzed using a GC-14A with a column temperature of 170 ℃, injection and detector temperatures of 200 ℃ and 220 ℃, respectively, and nitrogen was used as the carrier gas at a flow rate of 15 mL min^−1^.

All gas chromatographs were interfaced to Hewlett-Packard HP3395 integrators (Palo Alto, CA) for data collection. Reactants and products were quantified by their retention times by a calibration plot generated by adding known amounts of reactant or product to a reaction vial that contained PB (1 mL) without cells. The calibration plots contained at least 5 different concentrations of reactant or product, including at least one concentration above the maximal concentration detected in our experiments. All calibration plots were fitted by linear regression and had final r^2^ values of ≥0.98.

### IB- and DEE-dependent growth and oxidation assays

Batch cultures of ELW1 were grown in 160 mL glass serum bottles on IB or DEE as described above. The bottles were sealed and then ~0.24 mmol of substrate (IB or DEE) was added by syringe. Growth and substrate consumption were measured by GC (for gaseous-phase analytes) as described above at the indicated time points.

For biomass comparisons, batch cultures of ELW1 were grown in 700 mL glass serum bottles on IB or DEE, as described above. The bottles were sealed and then ~0.38, 0.76, or 1.14 mmol of substrate (IB or DEE) was added by syringe. Substrate consumption was measured by GC (gaseous-phase analytes) as described above. Cells were harvested by centrifugation when IB or DEE was no longer detected in the headspace in pre-weighed glass centrifuge tubes. After a single washing step with PB (20 mL), the centrifuge tubes with cell pellets were placed in an 85 ℃ incubator to dry. Biomass was calculated after three days of consistent measurements (<0.1% mass change) using a Sartorius A 210 P analytical scale (Sartorius Corporation, Bohemia, NY).

For detection of DEE oxidation and products, batch cultures of ELW1 were grown in 700 mL glass serum bottles on IB or DEE, as described above. The bottles were sealed and then IB (∼10%, v/v, gas phase) or DEE (1 mmol) was added by syringe. Cultures were grown to stationary phase (>1.0 OD_600_) and then were harvested, as described above. Aliquots (100 μL) of the concentrated cell suspension were added to glass serum vials (10 mL) containing PB (900 μL) to give a final reaction volume of 1 mL and a protein concentration between ∼0.75 and 2 mg total cell protein mL^−1^. Cells were treated with 1-octyne (1 µmol of a 5 M stock solution in DMSO) or with (9 µmol neat DMSO), sealed with butyl rubber stoppers and aluminum crimp caps, and incubated in a shaking water bath (150 rpm; 1 h; 30 ℃). After incubation, the cells were washed three times in PB (1 mL), centrifuged (10,000 RCF; 3 min; RT), and then finally resuspended in PB (1 mL) and transferred to new glass serum vials (10 mL) which were then sealed and incubated in a water bath, as described previously. The analysis was initiated by the injection of neat DEE (0.5 µL [~ 5 µmol]) into the vials 1 min prior to the initial GC injection. Following GC analysis, the cells were harvested by centrifugation, and the protein concentration was determined, as described above.

To estimate the specific activity of isobutane oxidation to TBA (Kottegoda 2014), batch cultures of ELW1 were grown in glass serum bottles (700 mL) with DEE (0.5 mmol), as described in the Methods section. Cultures were grown to stationary phase (>1.0 OD_600_) and then were harvested, as described above. Aliquots (100 μL) of the concentrated cell suspension were added to glass serum vials (10 mL) containing PB (900 μL) to give a final reaction volume of 1 mL and a protein concentration of ∼0.6 mg total cell protein mL^−1^. The vials were sealed with butyl rubber stoppers and aluminum crimp seals. Isobutane (~10%, v/v, gas phase) was injected into the vial as an over-pressure to initiate the reaction. At the indicated time points the production of TBA was determined by GC analysis of the aqueous phase.

### ELW1 and ELW1ΔpELW1-1 growth assays

Growth was measured on batch cultures of ELW1 and a plasmidless strain, ELW1ΔpELW1-1, on IB (10% v/v, gas phase), DEE (0.125 mmol), ethanol (0.125 mmol), acetaldehyde (0.125 mmol), DEE (0.125 mmol) plus IB (~ 1% v/v, gas phase), or fructose (0.125 mmol) in 160 mL glass serum bottles, as described above. The bottles were inoculated to an initial OD_600_ of ~0.02 with either a suspension of ELW1 cells previously grown on MSM agar with isobutylene (∼10%, v/v, gas phase) or ELW1ΔpELW1-1 cells previously grown on PCA. Growth was measured at the indicated time points.

### RNA-seq

Batch cultures of strain ELW1 were grown on DEE (0.5 mmol) in glass serum bottles (700 mL), as described above. The bottles were inoculated to an initial OD_600_ of ~0.35 from washed cells previously grown in batch culture on fructose (1 mmol) to late log phase (~0.8 OD_600_). Separate aliquots of the fructose-grown cells were saved for RNA-seq to serve as a control. After growth on DEE for 96 h, the cells were harvested by centrifugation (10,000 RCF; 5 min; 4 ℃) and then pelleted to ~3.75 × 10^8^ cells per sample for RNA-seq. The cell pellets were immediately frozen in a dry ice and ethanol bath for 4 min and then stored at − 80 ℃ until used.

Cell pellets were then submitted to the North Carolina State Genomic Sciences Laboratory (Raleigh, NC, USA) for RNA extraction, ribosomal depletion, and Illumina RNA library construction and sequencing. The final quantified libraries were pooled in equimolar amounts for clustering and sequencing on an Illumina NextSeq 2000 DNA sequencer, utilizing a P4 150 PE XLEAP flow cell and sequencing reagent kit (Illumina, USA). Real Time Analysis (RTA) software package was used to generate raw bcl, or base call files, which were then de-multiplexed by sample into FASTQ files for data submission. CLC Genomics Workbench v25.0 (QIAGEN) was used as described in Table S1 to perform differential expression analysis. Reads were mapped to the ELW1 chromosome and plasmid (Accessions: CP193805.1 and CP193804.1). Differential expression analysis was performed using the initial fructose-grown inoculating cultures as the control to which all other growth conditions and incubation times were compared (sample sizes: fructose-grown inoculating culture, n = 3; DEE-grown, n = 3). Sequencing and mapping statistics for RNA-seq are in Table S2. A detailed protocol for RNA-seq raw data processing and analysis is in the Supplementary Information (Table S1). Raw data can be found at NCBI’s Sequence Read Archive (SRA) (Bioproject: PRJNA1421072, Biosample: SAMN48782278, Accessions: SRR37182428–SRR371822433).

### Growth on DEE with IB

Batch cultures of ELW1 were grown in glass serum bottles (160 mL), as described above. The bottles were inoculated with ELW1 cells to an initial OD_600_ of ~0.01. The bottles were sealed and then IB was added by syringe in the following quantities 0.0, 4.5, 11.2, 22.3, 33.5, 44.6, 67.0, or 89.3 µmol. DEE (0.25 mmol) was added by syringe to designated bottles. Growth was measured after 6 days for all samples.

### Activity-based labeling (ABL)

Batch cultures of ELW1 were grown on IB or DEE in glass serum bottles (700 mL), as described above. The bottles were sealed and then IB (∼10%, v/v, gas phase) or DEE (0.5 mmol) were added by syringe. Cultures grew to stationary phase (>1.0 OD_600_) and then were harvested by centrifugation (10,000 RCF; 5 min; 4 ℃), washed three times in PB (10 mL), and then finally resuspended in PB to ~4.0 OD_600_. For ABL analyses, aliquots (1 mL) from the harvested cell suspension were added to sealed serum vials (10 mL) and either DMSO alone (1 µmol) or a diyne probe (1 µmol of 1 M stock solutions in DMSO) was added. The vials were then incubated in a shaking water bath (150 rpm; 1 h; 30 ℃). After incubation, cells were harvested by centrifugation (10,000 RCF; 3 min; RT), washed three times with PB (1 mL), and then the protein concentration was determined, as described above. A sample (50 µg protein) from the cell lysate was then treated with Alexa Fluor™ 647 azide using a copper(I)-catalyzed alkyne–azide cycloaddition (CuAAC) (click reaction), as described previously (Bennett et al. 2016; Joyce and Hyman 2025). Subsequently, 5 µg of total protein per sample were separated by discontinuous SDS-PAGE (10% acrylamide gel) and then immediately imaged with a LICOR Odyssey® CLx System. AlexaFluor-labeled proteins were detected at 700 nm while total protein staining by Coomassie Brilliant Blue R-250 was detected at 800 nm. In both cases the resulting images were analyzed with Empiria Studio® Software (Image Studio™ Software v5.2).

### LC–MS/MS Identification of fluorescently labeled proteins

A batch culture of ELW1 was grown on DEE (0.125 mmol) in a glass serum bottle (160 mL) until late log phase (~ 0.8 OD_600_), as described above. The culture was split into two equal aliquots (12.5 mL). One sample was used for ABL analyses, as described above, while the other aliquot was untreated. Samples (5 µg total protein) from both aliquots were analyzed by SDS-PAGE using 10% Mini-PROTEAN® TGX™ Precast Gels (BIO RAD, USA). After SDS-PAGE, the gels were first visualized by fluorescent NIR and then stained with Coomassie Brilliant Blue R-250. The Coomassie stained gel was submitted to the UNC Metabolomics and Proteomics (MAP) Core where they excised the Coomassie-stained bands at the equivalent migration point of the observed fluorescently labeled bands. This was followed by in-gel tryptic digestion, LC–MS/MS analysis, and subsequent protein identification. The LC–MS/MS analysis used a Thermo UltiMate 3000-Exploris 480 and a 90-min Data Dependent Acquisition method. Data were searched in Proteome Discoverer (Thermo Scientific; v3.1) against the *Mycobacterium* sp. ELW1 reference proteome from Uniprot (https://www.uniprot.org/proteomes/UP000324606) (5,850 sequences) and a common contaminants database (245 sequences). Protein results were filtered for 1% FDR on the protein and peptide level and a minimum of 2 peptides identified.

## Results

### Growth of strain ELW1 on DEE

**Fig. 1:**
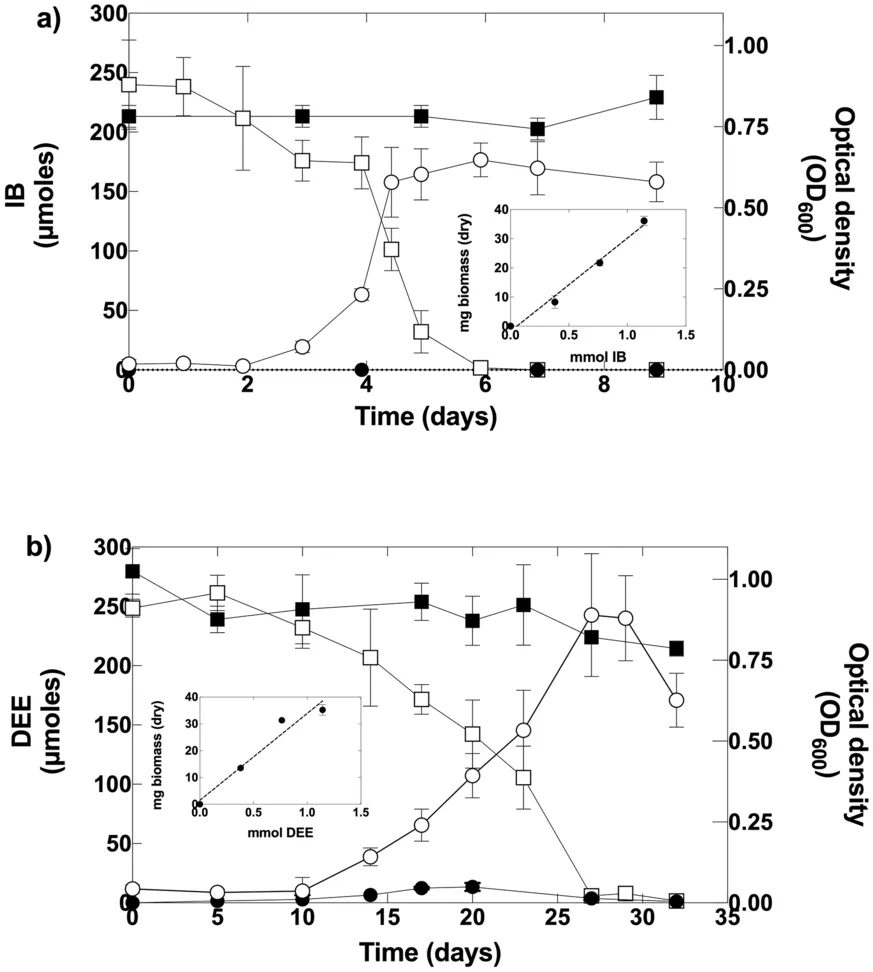
**Growth and consumption of IB and DEE by ELW1.** The Figure shows the time course of changes in culture density and substrate consumption by ELW1 cultures grown in 160 mL serum bottles on IB (Panel **a**) or DEE (Panel **b**) as sole sources of carbon and energy. In both Panels the square symbols represent substrate consumption in (■) uninoculated or (□) inoculated cultures while circular symbols represent culture turbidity (OD_600_) in the (●) absence or (○) presence of substrate. The insets in both Panels show plots of the dry whole-cell mass obtained after exhaustion of the indicated amount of substrate. The growth data presented are the means and SD obtained from biological replicates for IB-grown cells (n=3) and DEE-grown cells (n=2). The biomass yield data are the means and SD obtained from biological replicates for IB-grown cells (n=3) and DEE-grown cells (n=3)

Cells were grown on varying amounts of IB or DEE and the biomass generated was determined after complete substrate consumption, as determined by GC analysis of the reaction medium or headspace. In both cases plots of biomass generated *versus* the amount of substrate consumed were close to linear (r^2^ ≥0.95) and biomass yields of 0.49 ±0.09 mg/mg and 0.49 ±0.07 mg/mg were determined for IB and DEE, respectively (Fig. 1a and b**; **insets). The time courses of batch growth of ELW1 on equimolar amounts of DEE or IB (~240 µmol) were also compared (Fig. 1). With an estimated initial dissolved IB concentration of ~180 µM, the first detectable increases in culture density in IB cultures occurred after ~3 days and subsequent culture density changes were associated with corresponding decreases in the gas phase concentration of IB. No further changes in culture density were observed after 5 days once IB was consumed to below its detection limit (≥4.5 µmol IB) (Fig. 1a). With an estimated initial dissolved DEE concentration of ~7.1 mM, the first changes in culture density in DEE cultures were detected after a lag phase of ~14 days and the culture density continued to increase slowly as DEE was consumed (Fig. 1b). No further increases in culture density were observed after DEE was fully consumed after 27 days. The growth rates for cells grown on IB and DEE were estimated from these data at 0.05 h^−1^ and 0.008 h^−1^, respectively.

Growth of ELW1 on DEE also exhibited a substrate concentration optimum. Assuming full dissolution of DEE into the reaction medium, the fastest growth was observed with an initial DEE concentration of 5 mM. Like cultures containing 2.5 mM DEE, cultures grown on 5 mM DEE appeared to reach stationary phase within 14 days. As the initial DEE concentration increased above 5 mM, the cultures exhibited progressively slower rates of growth as well as a progressively increasing lag phases before growth was initiated. (Fig. S1).

**Fig. 2:**
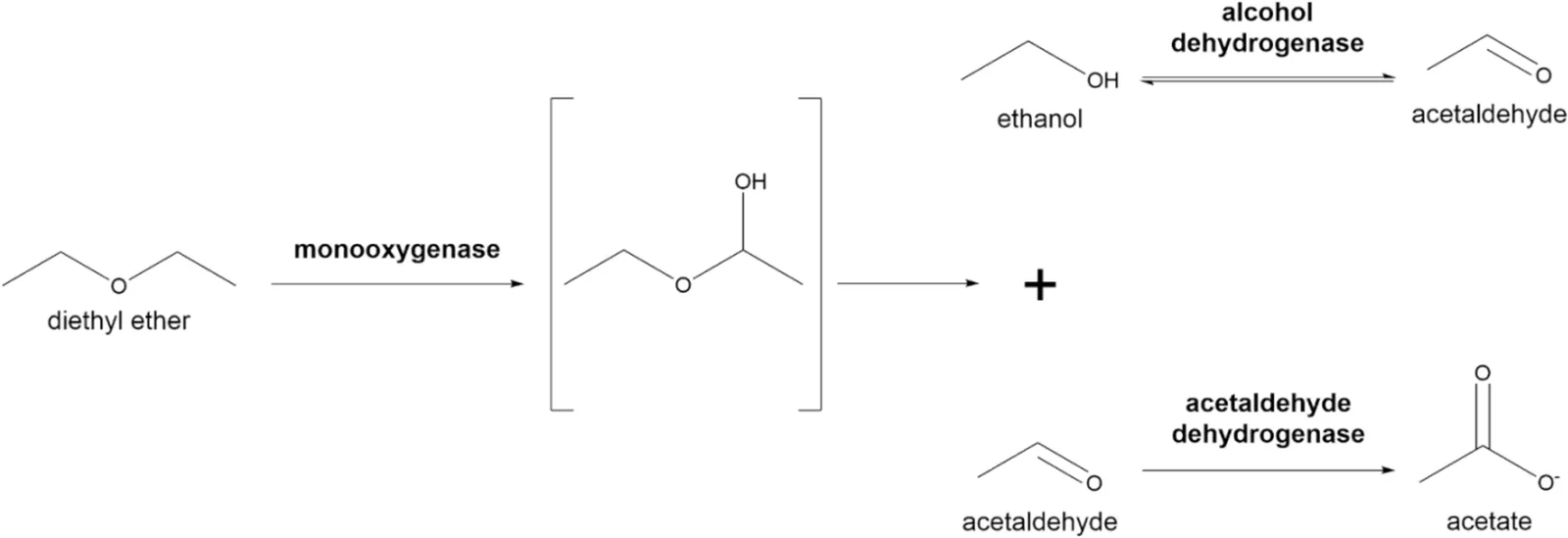
Proposed catabolic pathway of DEE by ELW1. The initial monooxygenase-catalyzed oxidation of one of the two ether bonded α-carbons of DEE results in a hemiacetal in which a single C atom is bonded to both a hydroxyl group and an ether group. The subsequent abiotic cleavage of the hemiacetal is acid catalyzed and involves protonation of the hydroxyl group, loss of that group as water, and subsequent release of equimolar amounts of ethanol and acetaldehyde (Hur et al. 1997; Kim and Engesser 2004). This cleavage is reversible, but the equilibrium strongly favors the products over the hemiacetal. Further oxidation of ethanol and acetaldehyde can be catalyzed sequentially by alcohol and aldehyde dehydrogenases

**Fig. 3:**
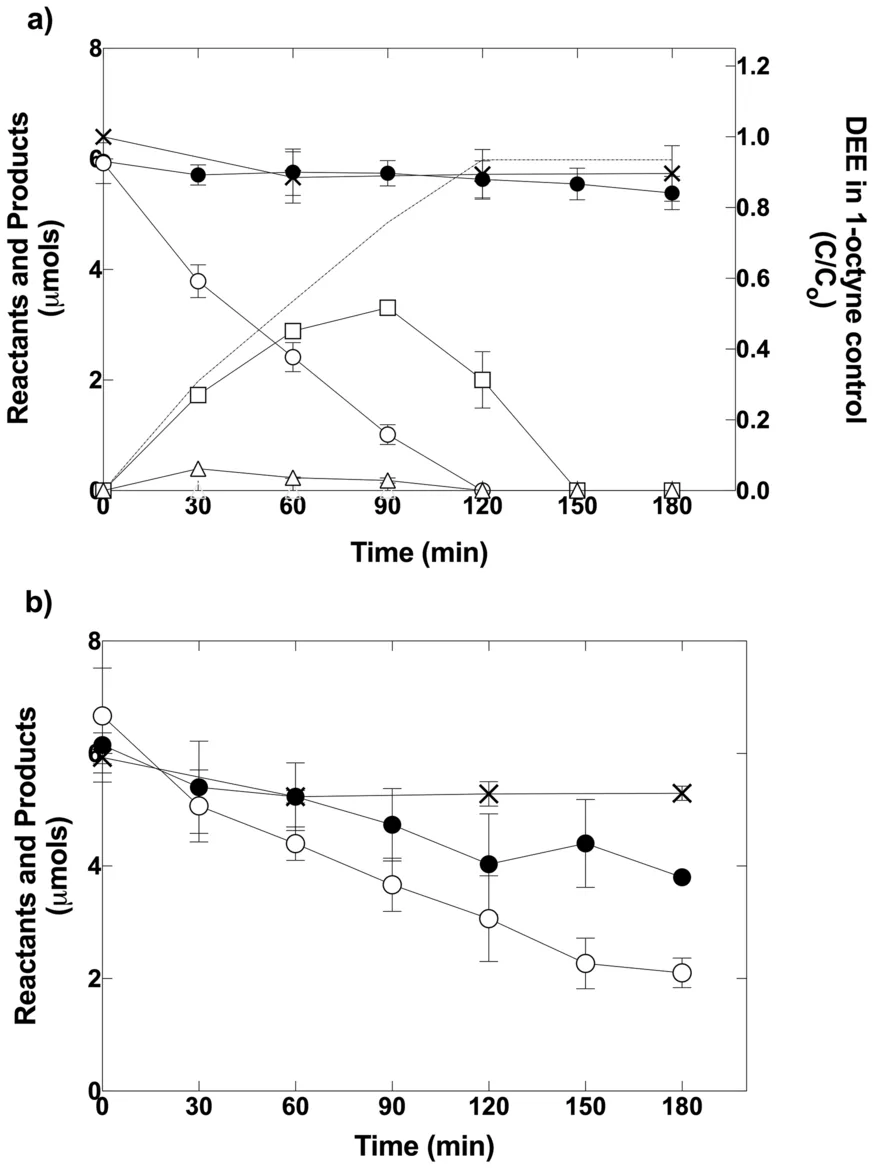
Oxidation of DEE by IB- and DEE-grown cells. The Figure shows the time course of DEE consumption and metabolite accumulation by resting cells previously grown on IB (Panel **a**) or DEE (Panel **b**). The symbols in the Figure show (X) DEE in uninoculated controls and (●, ○) DEE consumption and the subsequent production and consumption of (□) ethanol and (△) acetaldehyde by cells either pretreated with (closed symbols) or without (open symbols) 1-octyne. The dotted line in Panel **a** represents the theoretical 1:1 stoichiometric production of ethanol from DEE oxidation. Neither ethanol or acetaldehyde were detected in reactions involving either 1-octyne treated IB-grown cells, DEE-grown cells, or in abiotic DEE reactions (these data are not shown for clarity). The data presented are the means and ranges of values obtained from three biological replicates (n=3). Note: In **a**, the initial starting concentration of DEE in 1-octyne treated samples was unequal between replicates (~6 vs ~4 mM), so for clarity these data are presented in the form of C/Co

As summarized in the Introduction, previous studies have concluded aerobic microbial DEE catabolism is frequently initiated by a monooxygenase-catalyzed O-dealkylation reaction that leads to the production of equimolar amounts of ethanol and acetaldehyde (Fig. 2). To evaluate this as a potential mechanism of DEE oxidation by ELW1, the time course of the consumption of DEE and metabolite accumulation were compared for resting IB- and DEE-grown cells. IB-grown cells consumed DEE (20.7 ±0.3 nmol.min^−1^.mg protein^−1^) and both ethanol and acetaldehyde were detected in the reaction medium (Fig. 3a). However, the stoichiometries of ethanol and acetaldehyde accumulation with respect to DEE consumption were different and a mass balance for DEE consumption and accumulation of these metabolites was not achieved. For example, ethanol accumulated stoichiometrically with respect to DEE consumption only over the initial 30 min of the reaction and reached a maximum concentration of ~3.3 mM (~66% of potential production) after 90 min. Subsequently, the ethanol concentration declined, and it was completely consumed from the reaction shortly after DEE was exhausted from the medium. In contrast, the maximal detected concentration of acetaldehyde (~0.4 mM) was only a small fraction (≤12%) of the amount of ethanol detected at any time point and the maximum acetaldehyde concentration was observed earlier than for ethanol. Previously it has been shown in resting cell assays that 1-octyne is a highly selective inhibitor of IMO-catalyzed reactions (Kottegoda et al. 2015). In the presence of 1-octyne, no DEE consumption above abiotic losses was observed for IB-grown cells and neither ethanol nor acetaldehyde were detected in the reaction medium at any point during the reaction time course (Fig. 3a). These data strongly suggest that IMO has a role in initiating DEE catabolism.

The rate of DEE consumption by DEE-grown cells (11.8 ±1.6 nmol.min^−1^.mg protein^−1^) was ~50% of the rate observed for IB-grown cells (Fig. 3b) and 1-octyne only inhibited the rate of DEE consumption by ~50% (residual rate = 5.5 ±0.9 nmol DEE oxidizied.min^−1^.mg protein^−1^). Neither ethanol or acetaldehyde were detected in the reaction medium when DEE-grown cells oxidized DEE, either in the presence or absence of 1-octyne. To confirm that the differences in specific rates of DEE oxidation observed between IB- and DEE-grown cells were observed with other substrates, the whole cell specific activity of *tertiary-*butyl alcohol (TBA) production from the oxidation of isobutane (2-methylpropane) (Kottegoda 2014) was determined for DEE-grown cells at 4.1 ±0.4 nmol TBA nmol.min^−1^.mg protein^−1^. We have previously reported a specific activity of 15.8 ± 6.2 nmol TBA nmol.min^−1^.mg protein^−1^ for IB-grown cells of ELW1 (Joyce et al. 2026).

**Fig. 4:**
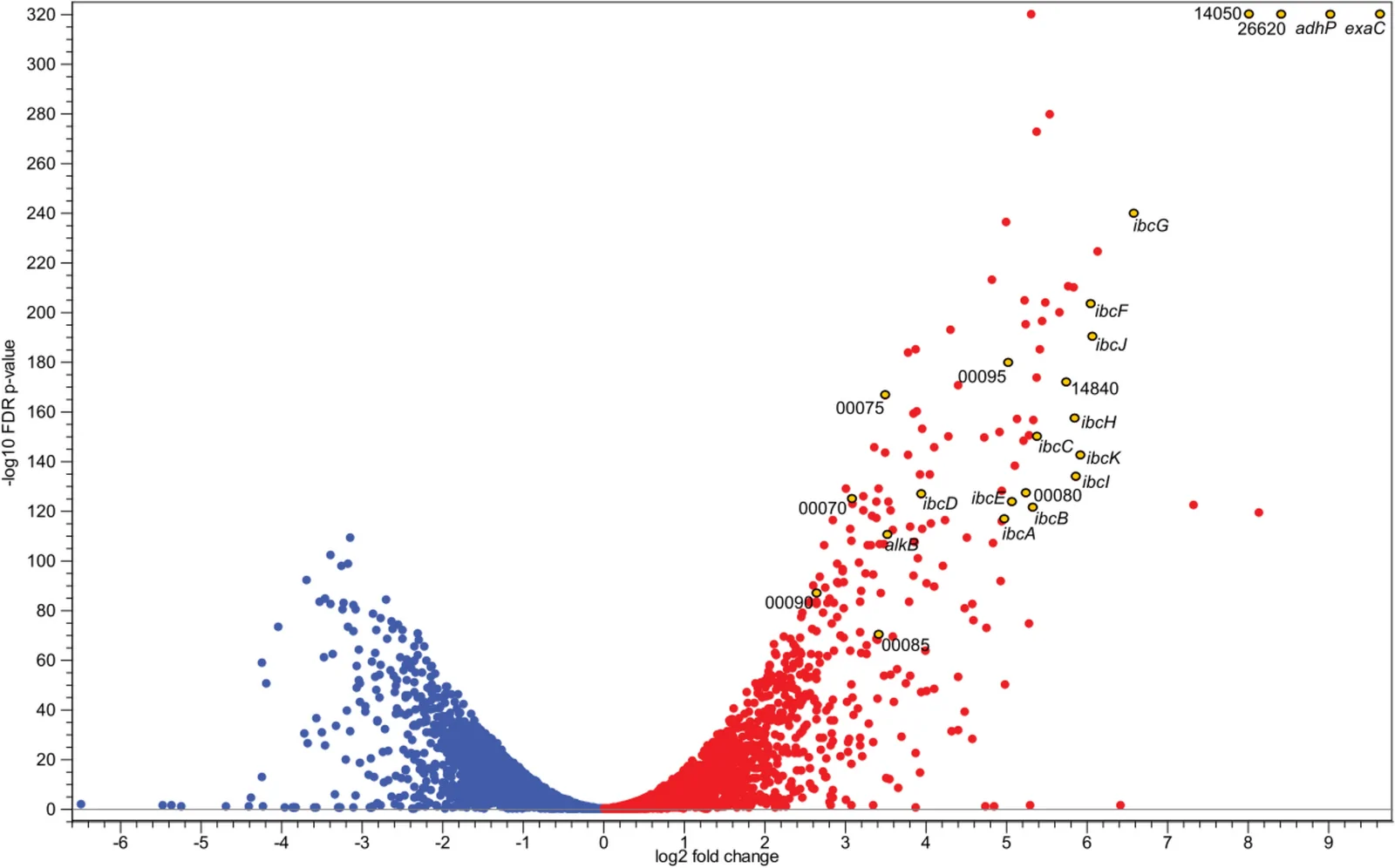
Volcano plot depicting the global transcriptional remodeling of DEE-grown ELW1 cells. The Figure shows the differentially expressed genes that were (red) upregulated or (blue) down regulated in DEE-grown cells compared to fructose-grown cells. Only genes with a |log2FC| value ≥1, adjusted p-value (FDR) ≤ 0.05, met our selection criteria and were significant for further analysis. Twenty-three genes of interest are labeled in the plot; details can be found in (Table S3)

**Differential gene expression in DEE-grown ELW1 cells**. To identify potential genes involved in DEE catabolism, RNA-seq and differential gene expression analyses were conducted that compared DEE-grown with fructose-grown cells. Of 6,202 transcripts identified by RNA-seq, 1,085 genes (16.95% of the chromosomal genes and 33.5% of the plasmid borne genes) were significantly upregulated and 1,137 genes (17.1% of the chromosomal genes and 6.8% of the plasmid borne genes were downregulated (|log_2_FC| value ≥1; adjusted p-value (FDR) ≤0.05) (Fig. 4). A full compilation of the differential expression analysis is deposited at Zenodo (https://doi.org/10.5281/zenodo.18805863).

Based on gene annotation and gene ontology function prediction, there are 46 aldehyde dehydrogenases and 22 alcohol dehydrogenases encoded by the ELW1 chromosome and two aldehyde dehydrogenases and one alcohol dehydrogenase encoded by the plasmid, pELW1-1. The genes with the highest overall upregulation (log_2_FC ≥8.0) in DEE-grown cells (Table S3) were a chromosomally-encoded alcohol dehydrogenase (ACRDU6_26420) closely related to AdhP (Thomas et al. 2013) (Fig. S2) and a chromosomally encoded acetaldehyde dehydrogenases (ACRDU6_26425) closely related to ExaC (Ko et al. 2025) (Fig. S3). These two genes are in a predicted operon that also contains a gene encoding an upregulated DNA-binding protein (ACRDUS_26430) (Taboada et al. 2018). In addition to another aldehyde dehydrogenase (ACRDU6_14840), other highly upregulated chromosomal genes encode two cytochrome P450s (ACRDU6_14050 and ACRDU6_26620), and an alkane 1-monoxygenase (*alkB*; ACRDU6_25345) (Table S3). Another 19 predicted monooxygenases including cytochrome P450s and flavin-containing enzymes were also upregulated, ranging in log_2_FC expression values from 1.01 to 2.43.

Among the upregulated genes in DEE-grown cells, 73 were located on the megaplasmid pELW1-1, and 10 of these genes (average log_2_FC = 5.67 ±0.48) are part of an isobutylene catabolic (*ibc*) gene cluster, *ibc* cluster 1, that encodes the enzymes (IbcA–K) putatively responsible for the catabolism of IB to 2-HIBA (Table S3) (Faulkner et al. 2025; Joyce et al. 2026). All the genes in an adjacent operon to *ibc* cluster 1 that encode a predicted Group 6 SDIMO were also upregulated (average log_2_FC = 3.49 ±0.31). In contrast to IB-grown cells (Joyce et al. 2026), the genes involved in the further transformation of 2-HIBA to central metabolic intermediates present in *ibc* cluster 2 were not upregulated (average log_2_FC = −0.95 ±1.11) in DEE-grown cells. Most of the remaining upregulated genes from pELW1-1 were hypothetical proteins and predicted mammalian cell entry (MCE) family proteins, which in microbial environmental strains, have been shown to have a role in lipid transport and in hypoxia, starvation, and oxidative stress responses (Klepp et al. 2022).

**Fig. 5:**
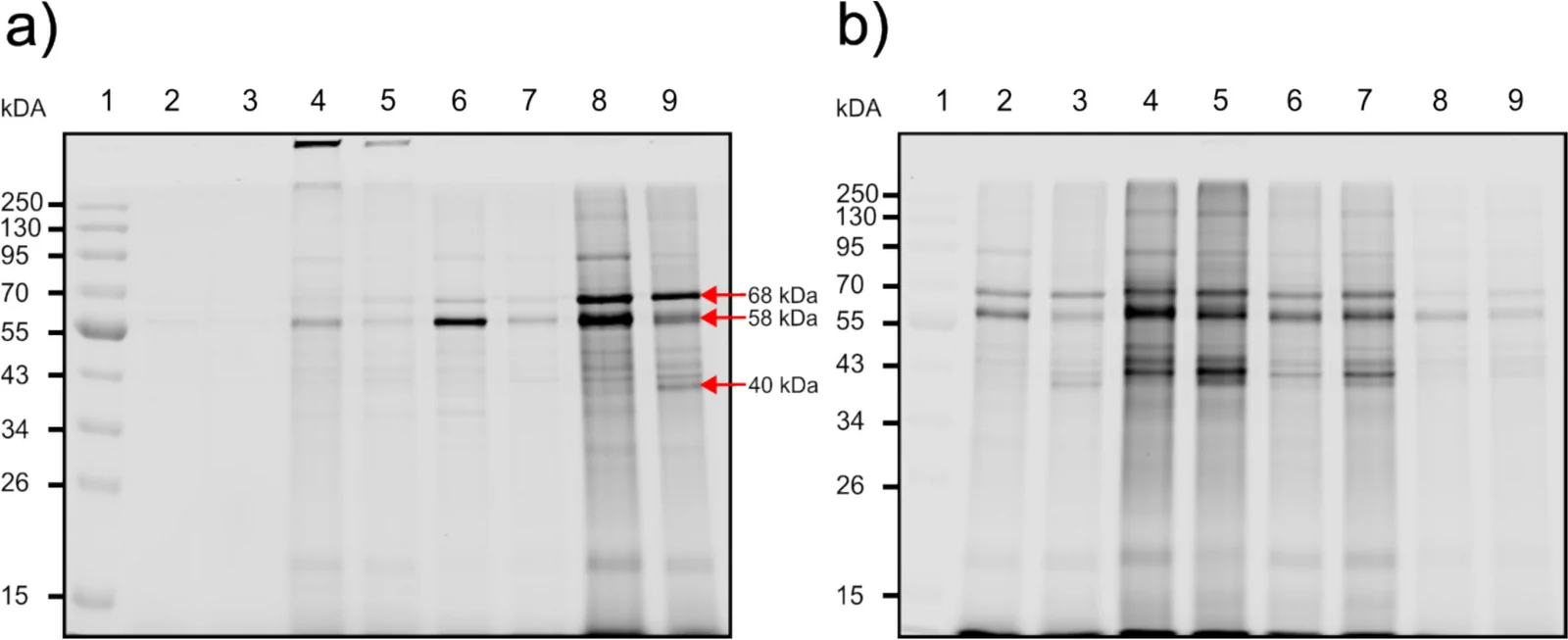
ABL analyses of IB- vs DEE-grown cells of ELW1. The Figure shows the fluorescent labeling patterns obtained following SDS-PAGE and NIR detection for IB- and DEE- grown cells exposed with different diyne ABL probes. In each panel, lanes 2, 4, 6, and 8 are soluble fraction protein samples from IB-grown cells while lanes 3, 5, 7, and 9 are soluble fraction cell protein samples from DEE-grown cells. For Panel **a**, the protein samples analyzed were: (Lane 1) PageRuler™ Prestained NIR Protein Ladder; or cells probed with either: (Lanes 2 & 3) DMSO (control); (Lanes 4 & 5) 1,4-diethynylbenzene (DEB); (Lanes 6 & 7) 1,5-hexadiyne (15HD); and (Lanes 8 & 9) 1,6-heptadiyne (16HD). For Panel **b**, the protein samples analyzed were: (Lane 1) PageRuler™ Prestained NIR Protein Ladder; or cells probed with either: (Lanes 2 & 3) 1,7-octadiyne (17OD); (Lanes 4 & 5) 1,8-nonadiyne (18ND); (Lanes 6 & 7) 1,9-decadiyne (19DD); and (Lanes 8 & 9) dipropargylamine (DIP). The red arrows indicate prominent fluorescent bands observed in both DEE- and IB-grown cells that were later excised for LC–MS/MS analysis

**Activity-Based Labeling (ABL) of IB- and DEE-Grown ELW1**. To further characterize the enzymes potentially involved in DEE catabolism, activity-based labeling (ABL) of diyne-sensitive monooxygenases was used to compare the fluorescent labeling patterns for cells from both IB- and DEE-grown cultures (Fig. 5) (Bennett et al. 2016; Joyce and Hyman 2025).

Although SDS-PAGE and NIR detection revealed different probes produced fluorescent bands with varying intensities, the fluorescent patterns were very similar for IB- and DEE-grown cells (Fig. 5) and two highly fluorescent bands with masses of ~58 kDa and ~68 kDa were detected for all probes examined. The ABL analysis of DEE-grown cells also showed the fluorescence associated with the ~58 kDa band was consistently lower for DEE-grown cells than IB-grown cells when treated with the same diyne ABL probe. This fluorescence ratio (IB:DEE) for the 58 kDa band varied from >7.5 to ~1 with a mean value of 2.97 (SD = 2.24) (Table S4). The kinetics of labeling were investigated for the ~58 kDa and the ~68 kDa fluorescent bands using 1,6 heptadiyne (16HD) as a probe (Fig. S4). Both bands were labeled at very similar rates though the labeling intensity of the 58 kDa band was consistently more intense than the 68 kDa band. A third fluorescent band with a mass of ~40 kDa was also detected in both IB- and DEE-grown cells and the labeling intensity of this band was maximal when cells were probed with 1,8-nonadiyne (18ND) (Fig. 5b; Lanes 4 and 5).

All three major fluorescent bands from DEE-grown cells were excised, subjected to in-gel tryptic digestion, and the resulting peptide mixtures were analyzed by UHPLC/mass spectrometry (Materials and Methods). Amino acid sequences for 466, 583, and 680 distinct proteins were obtained for the ~68 kDa, ~58 kDa, and ~40 kDa bands, respectively. The most abundant protein identified for the ~68 kDa band was IbcH, the putative MPD-oxidizing alcohol dehydrogenase (Table S5). For the ~58 kDa band, the most abundant proteins identified were the ExaC-like dehydrogenase, followed by IbcA, the α-subunit of IMO (Table S5). The most abundant proteins identified for the ~40 kDa band were IbcK, the putative IBO epoxide hydrolase involved in IB catabolism, followed by the AdhP-like dehydrogenase (Table S5). The Ibc enzymes identified in these bands are all encoded by genes in *ibc* cluster 1.

**The megaplasmid pELW1-1 is required for growth on DEE**. As extensive upregulation of *ibc* cluster 1 genes was observed for DEE-grown cells (Table S3), a plasmid-free strain of ELW1 (Joyce et al. 2026), designated as ELW1ΔpELW1-1 (GenBank accession CP194511), was used to investigate if growth of ELW1 on DEE and other substrates required plasmid-encoded enzymes. Wildtype ELW1 grew on IB (10% v/v gas phase), DEE (5 mM), ethanol (5 mM), and fructose (5 mM). Growth on DEE also increased substantially if cultures were incubated simultaneously with low levels of IB (~ 0.75% v/v gas phase) (Table 1). In contrast, strain ELW1ΔpELW1-1 only grew on ethanol and fructose and did not grow on DEE, either with or without supplemental IB (Table 1).

**Table 1:**
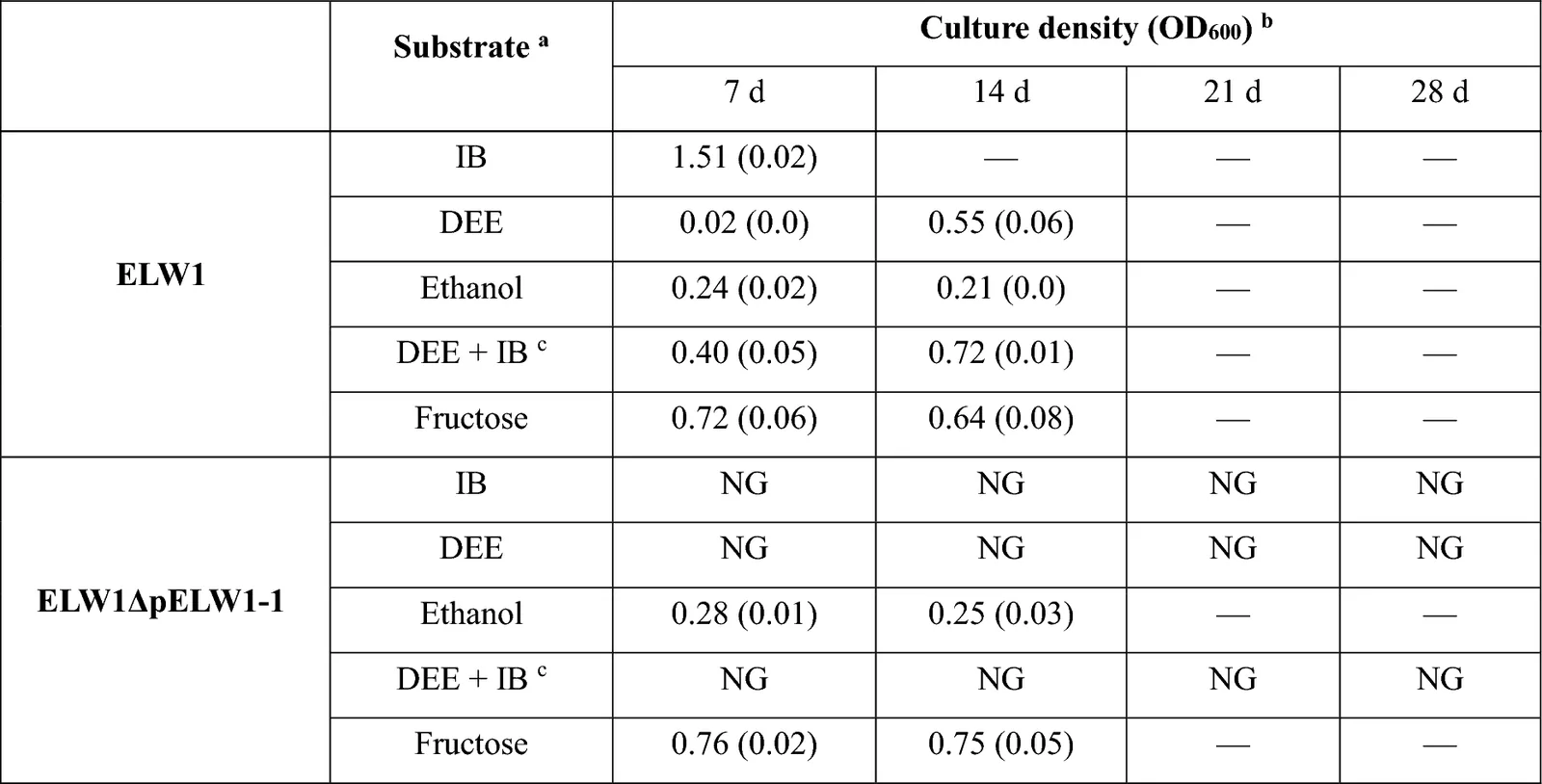
Growth substrate range of ELW1 and ELW1ΔpELW1

a^5^ mmol liquid or 10% (v/v) gaseous substrates were used in cultures. Filter-sterilized aqueous stock solutions (1 M) were used for all liquid substrates, except DEE which was added as a neat compound

^b^Initial culture OD600 ≤ 0.02. Data represents the mean and SD (in parentheses) of two biological replicates

^c^ ~0.75% (v/v) gaseous IB supplementation. No growth (NG, monitored for 28 days). Cultures monitored but measurements not taken (—)

**Fig. 6:**
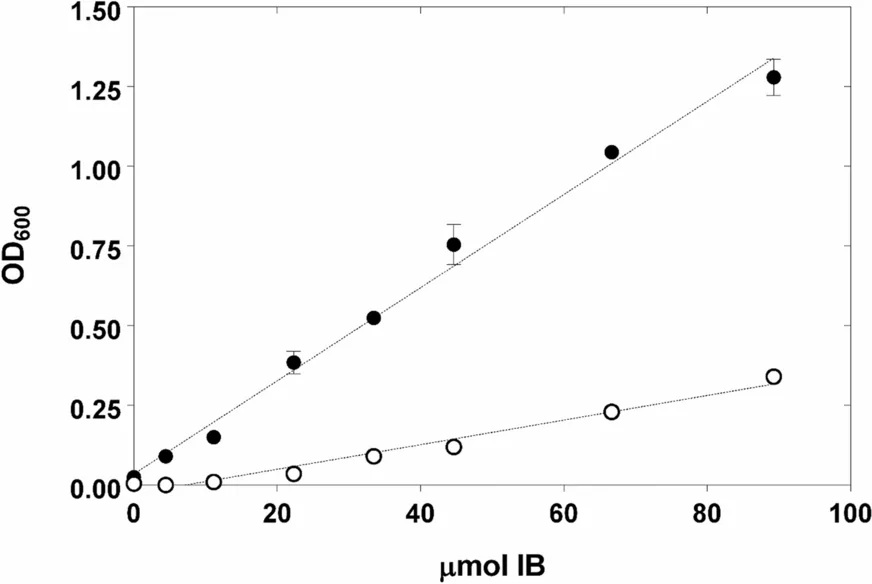
Isobutylene supplementation accelerates growth on DEE. The Figure shows the effect of including the indicated low levels of IB to ELW1 cultures growing on DEE (10 mM). The symbols show the corresponding culture densities measured after 6 days for cultures grown on (○) IB alone or (●) DEE supplemented with IB. The data presented are the mean and SD of two biological replicates. IB alone (slope = 0.0039 ± 0.00019; r^2^ = 0.97); DEE + IB (slope = 0.0146 ± 0.00042; r^2^ = 0.99)

**Effects of IB on DEE-utilizing cells**. The results in Table 1 indicate that the addition of small amounts of IB stimulated growth of ELW1 on DEE. This effect was further explored using cultures that contained a constant initial amount of DEE (10 mM (250 µmoles)) and varying initial amounts of IB (0 to 90 µmoles, 0 to 1.5% (v/v) gas phase). Compared to the limited growth observed for cultures containing IB alone, IB consistently increased the final culture density of DEE-containing cultures by an average of 3.7-fold in an IB concentration dependent manner (Fig. 6). These results are compatible with IB accelerating the rate of DEE oxidation. Confirmation of this was demonstrated by adding a low level of IB (≤1% v/v gas phase) to cultures containing cells that were initially grown on fructose, washed and then incubated with DEE (5 mM). During the initial 72 h of the incubation no changes in culture densities were observed reflecting the lag phase previously observed in this study (Fig. 1b). Following the addition of IB after 72 h, the culture densities in DEE-containing, IB-supplemented cultures increased ~59% over the next 24 h while the densities of cultures containing DEE alone only increased by ~16% over the next 24 h (Fig. S5).

The effect of adding compounds other than IB to DEE-containing cultures was also examined. The added compounds included other volatile alkenes, alkanes, and alkynes, as well as several established intermediates in the IB catabolic pathway. Out of the 16 different compounds tested, only DEE-containing cultures supplemented with either IB, 3-methyl-1-butene, or 2-HIBA exhibited increased growth compared to control cultures growing on DEE alone (Table S6).

## Discussion

In this study, we have demonstrated the likely pathway and enzymes involved in the catabolism of DEE by strain *Mycolicibacterium* sp. ELW1. The evidence strongly suggests growth on DEE relies on both plasmid-borne and chromosomal genes. Whole-cell assays, transcriptomic, and proteomic analyses indicated the primary monooxygenase most likely responsible for the initial oxidation of DEE is the plasmid-encoded IMO. Further transformation of the DEE metabolites ethanol and acetaldehyde likely involve AdhP- and ExaC-like chromosomally encoded dehydrogenases. The evidence supporting these conclusions is discussed in more detail in the following sections.

**DEE as a growth-supporting substrate for ELW1**. In this study ELW1 was shown to grow ~6-fold more slowly on DEE than on IB when cultures were grown in the presence of equimolar amounts of each substrate (Fig. 1). However, the Henry’s Law constants for DEE and IB (0.00125 and 0.63 atm.m^3^/mol, respectively) indicate that in this experiment the majority of DEE (73%) was dissolved in the aqueous phase compared to only ~2% of the added IB. Consequently, the results shown in Fig. 1 therefore compare growth rates for cells cultured with ~35-fold higher initial dissolved aqueous concentrations of DEE than IB. These differences in substrate distribution therefore make it difficult to meaningfully compare the respective growth rates supported by these substrates.

Unlike the previously characterized DEE-metabolizing strain *Rhodococcus* DEE5151, growth of ELW1 on DEE was also impacted by the initial culture DEE concentration and differences in initial DEE concentration had substantial impacts on both lag phases and culture biomass accumulation rates (Fig. S1). The cause of these effects is unclear but could potentially include several factors such as toxicity related to reactive peroxides in DEE itself or the accumulation of reactive metabolites such as acetaldehyde. Other possibilities include direct inhibition of enzyme activities by DEE (Aune et al. 1981), or membrane disrupting effects of DEE as a solvent (Bigelow and Thomas 1987). Despite these potentially detrimental effects, DEE supported an equivalent biomass yield to IB (Fig. 1).

**Role of monooxygenases in DEE catabolism**. In the present study the initial rate of DEE oxidation was ~2-fold faster in IB-grown than DEE-grown cells (Fig. 3). As IMO is highly expressed in IB-grown cells (Joyce et al. 2026), the higher rate of DEE oxidation observed with IB-grown cells suggests IMO is likely an important, if not the predominant catalyst for DEE oxidation in IB-grown cells. This conclusion is also supported by our observation that IB-grown cells also have as much as four-fold higher rates of IMO-catalyzed isobutane-dependent TBA production than DEE-grown cells. It is also important to note that DEE oxidation by IB-grown cells was fully inhibited by the IMO-selective inactivator, 1-octyne (Fig. 3a) (Kottegoda et al. 2015). In contrast, although DEE-grown cells were treated with 1-octyne under identical conditions to IB-grown cells, DEE oxidation by DEE-grown cells was only partially inhibited by this alkyne (Fig. 3b). This difference in sensitivity to 1-octyne suggests DEE oxidation in DEE-grown cells is catalyzed by at least one 1-octyne sensitive monooxygenase and one less 1-octyne sensitive monooxygenase. While the 1-octyne-sensitive enzyme is likely to be IMO, the potential identities of other DEE-oxidizing monooxygenases is less clear. An AlkB-containing alkane hydroxylase, several P450s, and Group 6 SDIMO were prominent among the monooxygenases upregulated in DEE-grown cells (Table S3). Some alkyne-sensitive AlkB-containing alkane hydroxylases can oxidize methyl ethers such as MTBE although the oxidation of ethyl ethers has not been reported for these enzymes (Smith and Hyman 2004, 2010; Katapodis et al 1988). The EthB-containing cytochrome P450 system catalyzes the O-dealkylation of simple ethers, including DEE (Schäfer et al. 2011) and diverse cytochrome P450s as well as Group 5 SDIMOs have been implicated in DEE oxidation in alkyl ether metabolizing *Rhodococccus* strains (Kim and Engesser 2004; Kim et al 2007). In the case of the predicted Group 6 SDIMO, we have previously shown that all subunits of this enzyme in IB-grown cells are upregulated at the transcript level but not at the protein level (Joyce et al. 2026) and this is also likely in DEE-grown cells. Supporting evidence for this conclusion is that the α-hydroxylase subunit of the Group 6 SDIMO has a predicted mass of ~59.1 kDa and is therefore expected to closely comigrate in SDS-PAGE with 58 kDa α-hydroxylase subunit of IMO (IbcA). Our proteomic analysis of the ~58 kDa fluorescent band (Table S5) demonstrated that for IB-grown cells the relative abundances for IbcA and the corresponding subunit from the predicted Group 6 SDIMO were 8.5 × 10^10^ and 1.19 × 10^8^, respectively while for DEE-grown cells these values were 1.7 × 10^11^ and 9.35 × 10^8^. In both cases these data suggest the active site-containing α-hydroxylase subunit of the Group 6 SDIMO is expressed at levels that are at least 2 orders of magnitude lower than IbcA and are therefore unlikely to contribute at any significant rate, if at all, to the observed rate of DEE oxidation in DEE-grown cells.

While further research will be needed to identify monooxygenases expressed by DEE-grown ELW1 that can O-dealkylate DEE but are either insensitive to inactivation by 1-octyne or are substantially less sensitive to 1-octyne than IMO, our observation that DEE does not support the growth of the plasmid-free strain ELW1ΔpELW1-1 is not incompatible with more than one monooxygenase contributing to the slow rate of DEE oxidation by DEE-grown cells. For instance, the rate of DEE oxidation by monooxygenases other than the plasmid-encoded and 1-octyne sensitive IMO may be too low to support growth of ELW1.

**Role of alcohol and aldehyde dehydrogenases in DEE catabolism**. Our transcriptomic analysis indicates genes encoding both an AdhP- and an ExaC-like alcohol and aldehyde dehydrogenase and the potential regulatory protein for this operon were among the most highly upregulated transcripts in DEE-grown cells (Table S3). This suggests that DEE-grown cells can rapidly oxidize ethanol and acetaldehyde. In support of this, rapid consumption of ethanol by DEE-grown cells was confirmed by whole-cell assays (Table S7). The lack of metabolite accumulation by resting DEE-grown cells during DEE oxidation (Fig. 3b) therefore likely reflects not only a relatively low rate of monooxygenase-catalyzed DEE oxidation but also the likelihood that the rate of ethanol and acetaldehyde oxidation by preexisting dehydrogenases exceeded their rate of monooxygenase-dependent production. Conversely, the time course and stoichiometry of metabolite accumulation during DEE oxidation by IB-grown cells (Fig. 3a) suggests both ethanol and acetaldehyde initially accumulated, albeit at different rates, during DEE oxidation but were then further oxidized. Our prior transcriptomic analysis indicates IB-grown cells expressed the AdhP-like and ExaC-like dehydrogenases at low levels compared to plasmid-borne *ibc* cluster 1 genes that encode alcohol (IbcH) and aldehyde (IbcG and IbcJ) dehydrogenases associated with IB and MPD catabolism (Joyce et al. 2026). This was also reflected in high rates of MPD oxidation and low rates of ethanol oxidation exhibited by IB-grown cells (Table S7). We therefore conclude that the low levels of ethanol and acetaldehyde-oxidizing activity in IB-grown cells can likely be initially attributed to previously expressed Ibc-related alcohol and aldehyde dehydrogenases, although accumulation of ethanol and acetaldehyde during DEE oxidation may have led to de novo expression of the AdhP- and ExaC-like dehydrogenases later in the reaction time course.

**Activity-based labeling of monooxygenases and other proteins**. The activity-based labeling approach used in this study is a powerful method for detecting and potentially quantifying catalytically active monooxygenases in complex protein samples (Bennett et al. 2016; Joyce and Hyman 2025). A key step in this method is the initial mechanism-based inactivation of target monooxygenases by diyne probes. An intrinsic limitation of using mechanism-based inactivators as ABL probes is that they can impact non-target proteins. This may be due to the diffusion of catalytically activated probes away from their immediate site of generation and the covalent modification of off-target proteins. Both the target and off-target proteins are then visualized in the second ABL step involving attachment of the fluorescent reporter group to covalently bound probe molecules. An analytical limitation of our approach is that denaturing 1D SDS-PAGE analyses of protein samples does not enable resolution of individual proteins but does resolve comigrating soluble proteins based on closely similar molecular weight. These two issues have important implications for the interpretation of the results of our ABL studies.

In this study the most abundant protein detected in the ~58 kDa fluorescent band was an ExaC-like aldehyde dehydrogenase (relative abundance [RA] = 1.80 × 10^11^) (Table S5) with a predicted mass of 54.9 kDa. The second, and equivalently abundant protein in the ~58 kDa band, was IbcA (RA = 1.70 × 10^11^). This 58 kDa protein is the active site-containing α-subunit of the hydroxylase component of IMO and the expected target of our ABL approach (Bennett et al. 2016; Joyce et al. 2026). Based on their respective monomeric molecular weights, these proteins should closely migrate in SDS-PAGE. As aldehyde dehydrogenases and none of the other major proteins detected in the ~58 kDa band are known to be inactivated by acetylenic compounds, we conclude that IbcA represents the large majority of the fluorescently labeled proteins in this band. Further strong support for this conclusion is provided by our previous shotgun proteomic analysis of the protein expression patterns of IB-grown cells of ELW1 (Joyce et al. 2026). This earlier study demonstrated the ExaC-like protein is present at very low levels in IB-grown cells while IbcA is present at very high levels. In the present study we observed very similar fluorescent labeling patterns for IB- and DEE-grown cells (Fig. 5) although our RNAseq data indicates very high levels of expression of the ExaC-like protein in DEE-grown cells.

In contrast, the identities of the labeled proteins in the ~68 and ~40 kDa fluorescent bands are less clear. The most abundant protein detected in the ~68 kDa band was the 59.8 kDa alcohol dehydrogenase, IbcH (RA = 1.55 × 10^11^). While IbcA was also among the 10 most abundant proteins detected in the ~68 kDa band, it was ~50-fold less abundant in the ~68 kDa band than IbcH. As none of the other 10 listed proteins detected in the ~68 kDa band are known to interact directly with alkynes, we conclude the fluorescence associated with this band is likely due to off-target labeling of IbcH although some alternative explanations exist. For example, IbcH could potentially undergo covalent modification as a result of a probe “metabolite” acting as a mechanism-based inactivator for IbcH. As an example of this, 3-butyn-1-ol and structurally similar acetylenic alcohols are mechanism based inactivators of alcohol dehydrogenases and the reactive species that covalently modifies the enzyme’s active site are aldehydes generated during oxidation of the alcohol group of these compounds (Alston et al. 1979). However, several lines of evidence suggest that off-target labeling is more likely. For instance, the fluorescent labeling of proteins in both the ~58 and ~68 kDa bands followed similar kinetics (Fig. S4) suggesting that both presumptively labeled proteins (IbcA and IbcH) are covalently modified by the same chemical species. Furthermore, exposure of both IB- and DEE-grown cells to 16HD, an inhibitor of IMO, only had a minor inhibitory effect on their rates of dehydrogenase-dependent IbcH-dependent MPD consumption (Table S7). This suggests the probe-dependent covalent modification of IbcH required for subsequent fluorescent labeling using ABL was not due to mechanism-based inactivation as it did not occur at the active site of the enzyme and result in enzyme inactivation. Co-A derivatives of acetylenic acids such as 2-pentynoyl-CoA are also potent mechanism-based inactivators of glutaryl-CoA and other acyl CoA dehydrogenases (Rao et al. 2003). While acyl-CoA dehydrogenases were also detected in both the ~68 and ~40 kDa fluorescent bands, like acetylenic alcohols, it is unclear how these potential inactivating species could be generated in vivo from simple diyne probes such as 16HD.

We also conclude that off-target labeling is the underlying cause of the more variable labeling observed for the ~40 kDa fluorescent band. In this band the most abundant detected protein (RA = 6.4 × 10^10^) was IbcK; the epoxide hydrolase responsible for transforming the initial epoxide intermediate in IB catabolism (Faulkner et al. 2025). IbcH (RA = 9.61 × 10^8^) and two acyl CoA-dehydrogenases were also detected (RAs = 1.8 × 10^9^ and 7.64 × 10^8^) while IbcA was not detected among the top 10 most abundant proteins. While IbcK could be susceptible to off-target labeling simply due to its overall high abundance in both IB and DEE-grown cells, it is also possible that it is preferentially labeled due to a close physical location to IMO. Aerobic alkene-oxidizing bacteria need to stringently control the concentration of the reactive and potentially toxic epoxides they generate during alkene catabolism and a close physical interaction between IMO and the IbcK epoxide hydrolase would facilitate this.

**Enzyme induction by DEE and stimulating effects of IB**. Previous studies have shown that DEE can be oxidized in vivo by several bacterial monooxygenases such as AMO, sMMO, and T2MO but, for varying reasons, DEE does not support growth of organisms expressing these different monooxygenases (Yilmaz et al. 1996; Hur et al. 1997; Schuster et al. 2013). While DEE-dependent induction is unlikely in the case of AMO and sMMO, it has been previously concluded that the inability of *B. vietnamiensis* to grow on DEE is due to the lack of T2MO induction by DEE (Hur et al. 1997). In this study we have presented multiple independent lines of evidence to suggest that growth on DEE induces expression of IMO and other *ibc*-related genes. For example, RNA-seq data for DEE-grown cells showed substantial transcriptional upregulation of IMO-encoding *ibc* cluster 1 genes compared to fructose-grown cells, and whole-cell enzyme assays using DEE-grown cells also demonstrated high rates of consumption of the IB catabolic intermediate, MPD (Table S7). Detection of IbcA by ABL also points to the presence of catalytically active IMO in DEE-grown cells. However, the slower rate of growth of ELW1 on DEE compared to IB (Fig. 1) and the slower rate of DEE oxidation by DEE-grown cells compared to IB-grown cells (Fig. 3) also point to an overall lower level of IMO activity in DEE-grown cells compared to IB-grown cells. This is further supported by the consistently observed higher amount of IMO detected by ABL (Fig. 5) in IB-grown cells and the lower rate of isobutane-dependent TBA production by DEE-grown vs IB-grown cells.

Another interesting question raised by our results (Fig. 6 and Table S6) is how small amounts of IB stimulate growth of ELW1 on DEE compared to DEE alone. The simplest explanation of this effect is that growth of ELW1 on the low concentrations of supplemental IB derives from accelerated rates of DEE oxidation and further metabolism of ethanol and acetaldehyde. Furthermore, as demonstrated in Fig S5, this effect is also manifested by a decrease in the lag phase prior to growth on DEE alone. At the mechanistic level, accelerated rates of DEE oxidation by preexisting IMO could potentially be due to IB-dependent alterations in the catalytic properties of IMO caused by the small regulatory protein in IMO (IbcD). Similar effects have been characterized with purified SDIMOs such as soluble methane monooxygenase (Walters et al. 1999) and isoprene monooxygenase (Sims et al. 2022). An alternative and more likely explanation is that IB upregulates overall expression of *ibc* cluster genes so that faster rates of oxidation of DEE and its metabolites can occur. The only other compounds that had a similar growth promoting effect on cultures growing on DEE were 3-methyl-1-butene and 2-HIBA (Table S6). While 3-methyl-1-butene may act through the same mechanism as IB, the purity of this compound used in this study was only 95%+ so it is possible that the observed stimulating effect is also due to minor contaminants. 3-methyl-1-butene (boiling point (bp) = 20.1 °C) is often commercially prepared by fractional distillation of mixed hydrocarbons generated from cracked crude oil and the principal contaminants are C_5_ alkenes and alkanes with similar boiling points Kaizik et al (2012). Several of these potential contaminants including 2-methyl-2-butene (bp = ~38 °C) and isoprene (bp = ~34 °C) were tested and shown to be unreactive in this study while other possible but untested contaminants include 2-methyl-1-butene (bp = ~31 °C), 1-pentene (bp = ~30 °C) and isopentane (bp = ~28 °C). In contrast, 2-HIBA is likely the inducer of *ibc* cluster 2 genes (cobalamin-dependent transformation of 2-HIBA ultimately to acetyl-CoA), but it may also regulate *ibc* cluster 1 in some way. Our prior studies using whole-cell enzyme assays and ABL suggest that growth on 2-HIBA alone does not upregulate IMO expression. Several other aerobic alkene-oxidizing bacteria use downstream intermediates as the inducers for monooxygenase expression. For example, in isoprene-utilizing strains, epoxyisoprene or another downstream intermediate acts as the primary inducer of *iso* gene expression, not isoprene itself (Crombie et al. 2015; Dawson et al. 2022). In *X. autotrophicus* Py2, in addition to gaseous alkenes, a range of chlorinated alkenes have been shown to induce expression of AMO (Small and Ensign 1997). If the effects of IB supplementation are due to enhanced expression of IMO and other genes in *ibc* cluster 1, it is notable that this effect is restricted to such a limited number of compounds. These intriguing observations warrant additional study into the regulation and expression of IMO, AdhP, and ExaC during DEE and IB-stimulated DEE catabolism in ELW1.

## Supplementary Information

Below is the link to the electronic supplementary material.

Supplementary file1 (DOCX 6899 KB)

## Data Availability

The results from LC–MS/MS identification of fluorescently labeled proteins can be found at https://doi.org/10.5281/zenodo.17780844. RNA-seq data are available, as described in Materials and Methods. A full dataset of the transcriptomic differential expression analysis can be found at (https://doi.org/10.5281/zenodo.18805863).

## References

[CR1] Allen JR, Clark DD, Krum JG, Ensign SA (1999) A role for coenzyme M (2-mercaptoethanesulfonic acid) in a bacterial pathway of aliphatic epoxide carboxylation. Proc Natl Acad Sci U S A 96:8432–8437 10.1073/pnas.96.15.8432 PMC17533 10411892

[CR2] Alston TA, Mela L, Bright HJ (1979) Inactivation of alcohol dehydrogenase by 3-butyn-1-ol. Arch Biochem Biophys 197:516–523. 10.1016/0003-9861(79)90275-3 507827

[CR3] Anthony C, Zatman L (1965) The microbial oxidation of methanol. The alcohol dehydrogenase of *Pseudomonas* sp. M27. Biochem J 96:808–812. 10.1042/bj0960808 PMC1207221 5862419

[CR4] Aune H, Stowell AR, Mørland J (1981) Ether inhibition of ethanol metabolism in isolated rat liver parenchymal cells. Alcohol Clin Exp Res 5:550–555. 10.1111/j.1530-0277.1981.tb05359.x 7030112

[CR5] Bennett K, Sadler NC, Wright AT et al (2016) Activity-based protein profiling of ammonia monooxygenase in *Nitrosomonas europaea*. Appl Environ Microbiol 82:2270–2279. 10.1128/AEM.03556-15 PMC4959501 26826234

[CR6] Bigelow DJ, Thomas DD (1987) Rotational dynamics of lipid and the Ca-ATPase in sarcoplasmic reticulum. The molecular basis of activation by diethyl ether. J Biol Chem 262:13449–13456 2820971

[CR7] Colby J, Stirling DI, Dalton H (1977) The soluble methane monooxygenase of *Methylococcus capsulatus* (Bath). Its ability to oxygenate *n*-alkanes, *n*-alkenes, ethers, and alicyclic, aromatic and heterocyclic compounds. Biochem J 165:395–402. 10.1042/bj1650395 PMC1164912 411486

[CR8] Coleman NV, Spain JC (2003) Distribution of the coenzyme M pathway of epoxide metabolism among ethene- and vinyl chloride-degrading *Mycobacterium* strains. Appl Environ Microbiol 69:6041–6046. 10.1128/AEM.69.10.6041-6046.2003 PMC201217 14532060

[CR9] Crombie AT, Khawand ME, Rhodius VA et al (2015) Regulation of plasmid-encoded isoprene metabolism in *Rhodococcus*, a representative of an important link in the global isoprene cycle. Environ Microbiol 17:3314–3329. 10.1111/1462-2920.12793 PMC4676930 25727256

[CR10] Dawson RA, Rix GD, Crombie AT, Murrell JC (2022) ’Omics-guided prediction of the pathway for metabolism of isoprene by *Variovorax* sp. WS11. Environ Microbiol 24:5151–5164. 10.1111/1462-2920.16149 PMC9804861 35920040

[CR11] Faulkner NW, Joyce JB, Smith C et al (2025) Characterization of an isobutylene epoxide hydrolase (IbcK) from the isobutylene-catabolizing bacterium *Mycolicibacterium* sp. ELW1. Appl Environ Microbiol 0:e00393-25. 10.1128/aem.00393-25 PMC12442371 40856435

[CR12] Hardison LK, Curry SS, Ciuffetti LM, Hyman MR (1997) Metabolism of diethyl ether and cometabolism of methyl *tert*-butyl ether by a filamentous fungus, a *Graphium* sp. Appl Environ Microbiol 63:3059–3067. 10.1128/aem.63.8.3059-3067.1997 PMC1389222 16535667

[CR13] Helbich S, Barrantes I, Dos Anjos Borges LG et al (2023) The 2-methylpropene degradation pathway in Mycobacteriaceae family strains. Environ Microbiol. 10.1111/1462-2920.16449 37321960

[CR14] Heydeman MT (1974) Growth of soil bacteria on diethyl ether. J Gen Microbiol 81:9–10

[CR15] Hur H, Newman LM, Wackett LP, Sadowsky MJ (1997) Toluene 2-monooxygenase-dependent growth of *Burkholderia cepacia* G4/PR1 on diethyl ether. Appl Environ Microbiol 63:1606–1609. 10.1128/aem.63.4.1606-1609.1997 PMC1389561 16535583

[CR16] Joyce JB, Faulkner NW, Hyman MR, Miller ES (2026) Aerobic catabolism of isobutylene by *Mycolicibacterium* sp. ELW1 requires inducible, plasmid-borne *ibc* genes. Appl Environ Microbiol 0:e02471-25. 10.1128/aem.02471-25 PMC13188929 41995308

[CR17] Joyce JB, Hyman MR (2025) Activity-based protein profiling of bacterial monooxygenases. In: Tao Z (ed) Activity-based proteomics: methods and protocols. Springer US, New York, NY, pp 293–317 10.1007/978-1-0716-4502-4_16 40515997

[CR18] Kaizik A, K-D Wiese, Maschmeyer D, Hess D, Bueschken W, Nierlich F (2012) Process for preparing 3-methyl-1-butene. US Patent 8,143,468 B2 27 Mar 2012

[CR19] Katapodis AG, Smith HA Jr, May SH (1988) New oxyfunctionalization capabilities for ω-hydroxylases: Asymmetric sulfoxidation and branched ether demethylation. J Am Chem Soc 110:897–899. 10.1021/ja00211a033

[CR20] Kim Y-H, Engesser K-H (2004) Degradation of alkyl ethers, aralkyl ethers, and dibenzyl ether by *Rhodococcus* sp. Strain DEE5151, isolated from diethyl ether-containing enrichment cultures. Appl Environ Microbiol 70:4398–4401. 10.1128/AEM.70.7.4398-4401.2004 PMC444782 15240329

[CR21] Kim Y-H, Engesser K-H, Kim S-J (2007) Physiological, numerical and molecular characterization of alkyl ether-utilizing rhodococci. Environ Microbiol 9:1497–1510 10.1111/j.1462-2920.2007.01269.x 17504487

[CR22] Klepp LI, y Garcia YS, FabianaBigi (2022) *Mycobacterial* MCE proteins as transporters that control lipid homeostasis of the cell wall. Tuberculosis. 10.1016/j.tube.2021.102162 34952299

[CR23] Ko JH, Jeong KH, Son SB, Lee JY (2025) Structural analysis of ExaC, an NAD^+^-dependent aldehyde dehydrogenase, from *Pseudomonas aeruginosa*. Biochem Biophys Res Commun 742:151077. 10.1016/j.bbrc.2024.151077 39642707

[CR24] Kohlweyer U, Thiemer B, Schräder T, Andreesen JR (2000) Tetrahydrofuran degradation by a newly isolated culture of *Pseudonocardia* sp. strain K1. FEMS Microbiol Lett 186:301–306. 10.1111/j.1574-6968.2000.tb09121.x 10802188

[CR25] Kottegoda WGSS (2014) Alkene and alkane oxidation by the 2-methylpropene-metabolizing Strain *Mycobacterium* sp. ELW1. North Carolina State University

[CR26] Kottegoda S, Waligora E, Hyman M (2015) Metabolism of 2-methylpropene (isobutylene) by the aerobic bacterium *Mycobacterium* sp. Strain ELW1. Appl Environ Microbiol 81:1966–1976. 10.1128/AEM.03103-14 PMC4345374 25576605

[CR27] Monticelli F (2014) Diethyl ether. Encycl Toxicol. 10.1016/B978-0-12-386454-3.00987-8

[CR28] Parales RE, Adamus JE, White N, May HD (1994) Degradation of 1,4-dioxane by an actinomycete in pure culture. Appl Environ Microbiol 60:4527–4530. 10.1128/aem.60.12.4527-4530.1994 PMC202014 7811088

[CR29] Rao KS, Albro M, Vockley J, Frerman FE (2003) Mechanism-based inactivation of human glutaryl-CoA dehydrogenase by 2-pentynoyl-CoA: rationale for enhanced reactivity*. J Biol Chem 278:26342–26350. 10.1074/jbc.M210781200 12716879

[CR30] Sales CM, Grostern A, Parales JV et al (2013) Oxidation of the cyclic ethers 1,4-dioxane and tetrahydrofuran by a monooxygenase in two *Pseudonocardia* species. Appl Environ Microbiol 79:7702–7708. 10.1128/AEM.02418-13 PMC3837796 24096414

[CR31] Schäfer F, Muzica L, Schuster J et al (2011) Formation of alkenes via degradation of *tert*-alkyl ethers and alcohols by *Aquincola tertiaricarbonis* L108 and *Methylibium* spp. Appl Environ Microbiol 77:5981–5987. 10.1128/AEM.00093-11 PMC3165420 21742915

[CR32] Schuster J, Purswani J, Breuer U et al (2013) Constitutive expression of the cytochrome P450 EthABCD monooxygenase system enables degradation of synthetic dialkyl ethers in *Aquincola tertiaricarbonis* L108. Appl Environ Microbiol 79:2321–2327. 10.1128/AEM.03348-12 PMC3623258 23354715

[CR33] Sims LP, Lockwood CWJ, Crombie AT et al (2022) Purification and characterization of the isoprene monooxygenase from *Rhodococcus* sp. Strain AD45. Appl Environ Microbiol 88:e00029-22. 10.1128/aem.00029-22 PMC9004368 35285709

[CR34] Small FJ, Ensign SA (1997) Alkene monooxygenase from *Xanthobacter* strain Py2. J Biol Chem 272:24913–24920. 10.1074/jbc.272.40.24913 9312093

[CR35] Smith CA, Hyman MR (2004) Oxidation of methyl *tert*-butyl ether by alkane hydroxylase in dicyclopropylketone-induced and *n*-octane-grown *Pseudomonas putida* GPo1. Appl Environ Microbiol 70:4544–4550. 10.1128/aem.70.8.4544-4550.2004 PMC492405 15294784

[CR36] Smith CA, Hyman MR (2010) Oxidation of gasoline oxygenates by closely related non-haem-iron alkane hydroxylases in *Pseudomonas mendocina* KR1 and other *n*-octane-utilizing *Pseudomonas* strains. Environ Microbiol Rep 2:426–432. 10.1111/j.1758-2229.2010.00155.x 23766116

[CR37] Taboada B, Estrada K, Ciria R, Merino E (2018) Operon-mapper: a web server for precise operon identification in bacterial and archaeal genomes. Bioinformatics 34:4118–4120. 10.1093/bioinformatics/bty496 PMC6247939 29931111

[CR38] Thomas LM, Harper AR, Miner WA et al (2013) Structure of *Escherichia coli* AdhP (ethanol-inducible dehydrogenase) with bound NAD. Acta Crystallogr Sect F Struct Biol Cryst Commun 69:730–732. 10.1107/S1744309113015170 PMC3702314 23832197

[CR39] van Hylckama Vlieg JE, Leemhuis H, Spelberg JH, Janssen DB (2000) Characterization of the gene cluster involved in isoprene metabolism in *Rhodococcus* sp. strain AD45. J Bacteriol 182:1956–1963. 10.1128/JB.182.7.1956-1963.2000 PMC101893 10715003

[CR40] van Hylckama Vlieg JET, Kingma J, Kruizinga W, Janssen DB (1999) Purification of a glutathione S-transferase and a glutathione conjugate-specific dehydrogenase involved in isoprene metabolism in *Rhodococcus* sp. Strain AD45. J Bacteriol 181:2094–2101. 10.1128/jb.181.7.2094-2101.1999 PMC93621 10094686

[CR41] Walters KJ, Gassner GT, Lippard SJ, Wagner G (1999) Structure of the soluble methane monooxygenase regulatory protein B. Proc Natl Acad Sci U S A 96:7877–7882. 10.1073/pnas.96.14.7877 PMC22155 10393915

[CR42] White GF, Russell NJ, Tidswell EC (1996) Bacterial scission of ether bonds. Microbiol Rev 60:216–232. 10.1128/mr.60.1.216-232.1996 PMC239425 8852901

[CR43] Yilmaz E, Curry S, Ciuffetti L, Hyman M (1996) Microbial degradation of alkyl ethers. In: Abstracts of the 96th General Meeting of the American Society for Microbiology. American Society for Microbiology, Washington, DC

