# Supplementary material for: Catabolism of diethyl ether as a sole carbon and energy source by Mycolicibacterium sp. ELW1
Source: Biodegradation. 2026 Oct 6;37(5):159. doi: 10.1007/s10532-026-10378-8 (PMC13642445; doi:10.1007/s10532-026-10378-8)
Supplement: Supplementary file 1 [file 10532_2026_10378_MOESM1_ESM.docx]

**Supplemental Material**

**Catabolism of diethyl ether as a sole carbon and energy source by *Mycolicibacterium* sp. ELW1**

**Authors:**

John B. Joyce^1^, Eric S. Miller^1,#^, and Michael R. Hyman^1,#^

**Address:**

^1^Department of Plant and Microbial Biology, North Carolina State University, Raleigh, North Carolina 27695-7615, USA

^#^Corresponding Authors: [eric_miller@ncsu.edu](mailto:eric_miller@ncsu.edu); [michael_hyman@ncsu.edu](mailto:michael_hyman@ncsu.edu)

**Table S1. CLC RNA-seq pipeline**

**Importing Illumina reads from NextSeq 2000 data with CLC Genomics Workbench v25.0 (QIAGEN)**

1. Illumina High-Throughput Sequencing Import
   a. Files per sample
      i. R1
      ii. R2
   b. Joined reads from multiple lanes
2. General Options (checked)
   a. Paired reads
   b. Discard read names
   c. Discard quality scores
3. Paired read information (checked)
   a. Paired-read (forward-reverse)
   b. Default min distance 1 and max distance 1000
4. Illumina options (checked)
   a. Remove failed reads
   b. Quality scores
      i. NCBI/Sanger or Illumina pipeline 1.8 and later
5. Result handling
   a. Save
   b. Create subfolders
   c. Create log

**QC for Sequencing Reads**

1. Prepare Sequencing Data
   a. QC for Sequencing Reads
2. Result handling
   a. Create graphical report (checked)
   b. Create supplementary report (checked)
   c. Save

**Trimming reads**

1. Prepare Sequencing data
   a. Trim reads
      i. Select imported paired reads
   b. Quality trimming (checked)
      i. Trim using quality scores
         1. Quality limit 0.05
      ii. Trim ambiguous nucleotides
          1. Max number = 2
   c. Adapter Trimming (checked)
      i. Automatic read-through adapter trimming
   d. Homopolymer trimming
      i. None checked
   e. Sequence trimming
      i. Defaults
   f. Sequence filtering
      i. Defaults
   g. Result handling (checked)
      i. Create report
      ii. Save
      iii. Crate log

**Read Mapping and RNA-seq and Small RNA Analysis**

1. RNA-Seq Tools
   a. RNA-Seq Analysis (**must use version 23.0.4 or beyond**)
      i. Select paired, trimmed reads from previous section
   b. Reference Settings (checked)
      i. Genome annotated with genes only
         1. Ref sequence ELW1 Reference Genome (Genome)
            a. From NCBI reference download tool on CLC
            b. CP193805.1 and CP193804.1 used
         2. Gene track
            a. Created with the ELW1 reference genome using CLC
         3. Do not use spike-in controls
   c. Mapping settings
      i. All default including Maximum number of hits for a read
         1. This was set to 10
            a. 2 may have worked just as well
            b. Previous settings of 1 resulted in unmapped reads due to duplicate copies of genes in genome, and the subsequent discarding of them by the program
   d. Expression Settings
      i. Strand setting
         1. Both
      ii. Library type setting
          1. Bulk
      iii. Expression level
           1. RPKM
   e. Output settings
      i. Fusion gene table settings
         1. 5
   f. Results Handling (checked)
      i. Create reads track
      ii. Create report
      iii. Create fusion gene table
      iv. Create list of unmapped reads

**Quality Control**

1. QC for Read Mapping
   a. Select mapped reads
2. Result handling
   a. Output options
      i. Create separate table with statistics for each mapping (checked)
      ii. Save

**Differential expression in two groups**

1. Select expression sample
   a. Selected all of IB samples using the (GE) track
2. Control group expressions
   a. Selected all of the Fructose samples
3. ConFig. normalization method (checked)
   a. Whole transcriptome RNA-Seq
   b. All other defaults
4. ConFig. filtering and outliers (checked)
   a. Filter on average expression for FDR correction
5. Result handling (checked)
   a. Save
   b. Create log

**Table S2. RNA-seq sequencing and mapping statistics.**

| **Sample Name** | **Number of Reads** | **Reads mapped in pairs (%)** | **Reads not mapped (%)** |
| --- | --- | --- | --- |
| D021 | 77,578,870 | 98.93 | 0.62 |
| D022 | 67,290,206 | 98.76 | 0.77 |
| D023 | 72,010,838 | 98.11 | 1.46 |
| F001 | 47,435,104 | 98.05 | 1.37 |
| F002 | 59,734,158 | 97.89 | 1.66 |
| F003 | 55,255,556 | 98.07 | 1.41 |

CLC Genomics Workbench v25.0. (QIAGEN) RNA-seq pipeline was used. D0 = DEE-grown cells of ELW1. F0 = Fructose-grown cells of ELW1.

**Fig. S1. The effect of DEE concentration on growth by *Mycolicibacterium* sp. ELW1.** The Figure shows the growth (OD_600_) of DEE-grown ELW1 cells on various concentrations of DEE. The concentration of DEE is represented by the following symbols: (⭘) 1 mM, (⬤) 2.5 mM, (⏹) 5 mM, (□) 10 mM, (▲) 25 mM, (△) 50 mM. Batch cultures of ELW1 were grown in glass serum bottles 160 mL, as described in the Methods section. The bottles were inoculated with ELW1 cells to an initial OD_600_ of ~0.01. Growth was measured at the indicated times. Data represents the mean and SD of two biological replicates.

**Table S3. Highly upregulated genes potentially involved in DEE catabolism.**

| **Genome location** | **Genome locus** | **Gene name** | **Gene description** | **log2FC** |
| --- | --- | --- | --- | --- |
| Chromosome | ACRDU6_26425 | *exaC* | acetaldehyde dehydrogenase | 9.64 |
| Chromosome | ACRDU6_26420 | *adhP* | alcohol dehydrogenase | 9.02 |
| Chromosome | ACRDU6_14050 |  | cytochrome P450 | 8.40 |
| Chromosome | ACRDU6_26620 |  | cytochrome P450 | 8.01 |
| Chromosome | ACRDU6_14840 |  | aldehyde dehydrogenase | 5.75 |
| Chromosome | ACRDU6_25345 | *alkB* | alkane 1-monooxygenase | 3.52 |
| Plasmid | ACRDU6_00010 | *ibcA* | IMO 𝛼-hydroxylase subunit | 4.97 |
| Plasmid | ACRDU6_00015 | *ibcB* | IMO 𝛾-hydroxylase subunit | 5.33 |
| Plasmid | ACRDU6_00020 | *ibcC* | IMO Riske-type ferredoxin | 5.38 |
| Plasmid | ACRDU6_00025 | *ibcD* | IMO coupling protein | 5.24 |
| Plasmid | ACRDU6_00030 | *ibcE* | IMO 𝛽-hydroxylase subunit | 5.07 |
| Plasmid | ACRDU6_00035 | *ibcF* | IMO reductase | 6.05 |
| Plasmid | ACRDU6_00040 | *ibcG* | aldehyde dehydrogenase | 6.58 |
| Plasmid | ACRDU6_00045 | *ibcH* | alcohol dehydrogenase | 5.85 |
| Plasmid | ACRDU6_00050 | *ibcI* | hypothetical protein | 5.86 |

**Table S3 (continued).**

| **Genome location** | **Genome loci** | **Gene name** | **Gene description** | **log2FC** |
| --- | --- | --- | --- | --- |
| Plasmid | ACRDU6_00055 | *ibcJ* | aldehyde dehydrogenase | 6.07 |
| Plasmid | ACRDU6_00060 | *ibcK* | epoxide hydrolase | 5.91 |
| Plasmid | ACRDU6_00070 |  | PMO reductase | 3.08 |
| Plasmid | ACRDU6_00075 |  | PMO coupling protein | 3.50 |
| Plasmid | ACRDU6_00080 |  | PMO 𝛽-hydroxylase subunit | 3.95 |
| Plasmid | ACRDU6_00085 |  | PMO 𝛼-hydroxylase subunit | 3.42 |
| Plasmid | ACRDU6_00090 |  | response regulator | 2.65 |
| Plasmid | ACRDU6_00095 |  | sensor kinase | 5.03 |

**Fig. S2. Evolutionary relationships of alcohol dehydrogenases (AdhP).** The predicted alcohol dehydrogenase (AdhP; XUV46162.1) from *Mycolicibacterium sp*. ELW1 shares a 47.63% amino acid identity (query cover = 95%; e-value = 3e-97) to AdhP from *Escherichia coli* K-12, an ethanol-inducible dehydrogenase with solved crystal structure (Thomas et al. 2013). Amino acid sequences were compared using NCBI Protein BLAST (Altschul et al. 1997; Camacho et al. 2009). Evolutionary analyses were conducted in MEGA12 (Kumar et al. 2024). The phylogeny was inferred using the Maximum Likelihood method and Jones-Taylor-Thorton (1992) model (Jones et al. 1992). The trees are drawn to scale, with branch lengths in the same units as those of the evolutionary distances used to infer the phylogenetic tree. * confirmed and ** postulated ethanol dehydrogenation.

**Fig. S3. Evolutionary relationships of acetaldehyde dehydrogenases (ExaC)****.** The predicted alcohol dehydrogenase (ExaC; XUV46162.1) from *Mycolicibacterium sp*. ELW1 shares a 64.55% amino acid identity (query cover = 100%; e-value = 0.0) to ExaC from *Pseudomonas aeruginosa* PA01, an ethanol-catabolizing bacterium with solved crystal structure for an NAD^+^-dependent aldehyde dehydrogenase (Ko et al. 2025). Amino acid sequences were compared using NCBI Protein BLAST (Altschul et al. 1997; Camacho et al. 2009). Evolutionary analyses were conducted in MEGA12 (Kumar et al. 2024). The phylogeny was inferred using the Maximum Likelihood method and Jones-Taylor-Thorton (1992) model (Jones et al. 1992). The trees are drawn to scale, with branch lengths in the same units as those of the evolutionary distances used to infer the phylogenetic tree. * confirmed and ** postulated ethanol dehydrogenation.

**Table S4. Measurements of fluorescence of the ~58 kDa band in IB- vs DEE-grown cells of ELW1 using different diyne probes in ABL.**

|  | **Fluorescent signal (a.u.) ^a^** | | | | | | | |
| --- | --- | --- | --- | --- | --- | --- | --- | --- |
|  | **DMSO** | **DEB** | **15HD** | **16HD** | **17OD** | **18ND** | **19DD** | **DIP** |
| **IB-grown** | 168 | 1380 | 6850 | 11000 | 17100 | 36200 | 19600 | 6350 |
| **DEE-grown** | 85.2 | 422 | 896 | 3080 | 6890 | 21100 | 20900 | 3840 |

^a^ Fluorescence signal intensity was measured in arbitrary units (a.u.) by LI-COR Image Studio™ v.5.2 Software.

Activity-based probes: 1,4-diethynylbenzene (DEB); 1,5-hexadiyne (15HD); 1,6-heptadiyne (16HD); 1,7-octadiyne (17OD); 1,8-nonadiyne (18ND); 1,9-decadiyne (19DD); and dipropargylamine (DIP). Dimethyl sulfoxide (DMSO) was used as a control and carrier medium. SDS-PAGE gels were scanned immediately after electrophoresis using a LI-COR Odyssey CLx Imager. Fluorescent protein bands were then visualized and measured using LI-COR Image Studio™ v.5.2 Software. With the 700 nm detection channel selected, the selected band’s fluorescent signal intensity was measured automatically using the Analysis tool (Add Rectangle) centered on the fluorescent band. Background was subtracted using the Median function; Border (all); Border width (3). 16HD was used primarily in the study due to its reliability and low background labeling.

**Fig. S4. Time course of changes in fluorescent signal for ~58 kDa and ~68 kDa bands.** The Figure shows the time-dependent changes in fluorescence associated with the ~58 and ~68 kDa bands for cells exposed to 1,6-heptadiyne (16HD). Batch cultures of ELW1 were grown on IB (10% v/v), washed, and resuspended in PB, as described in the Materials and Methods of the main text for activity-based labeling (ABL). Resting cell suspensions were then incubated with 0.1 µmol 1,6-heptadiyne (16HD). At the indicated times cells were washed, lysed, and protein samples were subjected to copper(I)-catalyzed alkyne–azide cycloaddition (CuAAC) (click reaction) and SDS-PAGE and NIR detection, as described in the Materials and Methods section in the main text. The fluorescence associated with the (●) ~58 and (■) ~68 kDa fluorescent bands were then visualized and measured using LI-COR Image Studio™ v.5.2 Software. With the 700 nm detection channel selected, the selected band’s fluorescent signal intensity was measured automatically using the Analysis tool (Add Rectangle) centered on the fluorescent band. Background was subtracted using the Median function; Border (all); Border width (3). Michaelis–Menten curve fitting was performed using GraphPad Prism v.6.04 for Windows, GraphPad Software, Boston, Massachusetts USA, www.graphpad.com. The data represent the means of two biological replicates.

**Table S5. Quantitative proteomic analysis (LC-MS/MS) of the most abundant proteins (10) identified by ABL and SDS-PAGE/NIR from soluble fractions of DEE-grown cells of ELW1.**

| **Band ID** | **Total proteins identified** | **Protein ID ^a^** | **Gene description** | **Coverage (%)** | **# PSMs ^b^** | **MW (kDa)** | **Abundances (Normalized)** |
| --- | --- | --- | --- | --- | --- | --- | --- |
| ~68 kDa Band | 466 proteins identified (12 contaminants, primarily keratins) | QEN17568 | *ibcH* | 99 | 913 | 59.8 | 1.55E+11 |
|  |  | QEN12438 | chaperonin GroEL | 97 | 605 | 56.5 | 8.81E+10 |
|  |  | QEN13284 | ATP synthase subunit alpha | 81 | 197 | 58.7 | 1.83E+10 |
|  |  | QEN12120 | acyl-CoA dehydrogenase | 78 | 148 | 66.5 | 7.62E+09 |
|  |  | QEN13959 | 30S ribosomal subunit | 84 | 139 | 53.4 | 4.73E+09 |
|  |  | QEN12098 | phosphoenol- pyruvate carboxykinase | 87 | 150 | 66.8 | 3.98E+09 |
|  |  | QEN17701 | *ibcA* | 63 | 79 | 58 | 3.19E+09 |
|  |  | QEN15753 | aldehyde dehydrogenase | 77 | 73 | 54.9 | 3.05E+09 |
|  |  | QEN14620 | transketolase | 73 | 112 | 75.5 | 2.61E+09 |
|  |  | QEN15587 | acyl-CoA carboxylase subunit beta | 77 | 107 | 58.4 | 2.44E+09 |

**Table S5 (continued).**

| **Band ID** | **Total proteins identified** | **Protein ID ^a^** | **Gene description** | **Coverage (%)** | **# PSMs ^b^** | **MW (kDa)** | **Abundances (Normalized)** |
| --- | --- | --- | --- | --- | --- | --- | --- |
| ~58 kDa  Band | 583 proteins identified (10 contaminants, primarily keratins) | QEN15753 | *exaC* | 89 | 736 | 54.9 | 1.80E+11 |
|  |  | QEN17701 | *ibcA* | 81 | 725 | 58 | 1.70E+11 |
|  |  | QEN17570 | *ibcJ* | 77 | 288 | 52.1 | 5.60E+10 |
|  |  | QEN13286 | ATP synthase subunit beta | 90 | 150 | 51.7 | 1.43E+10 |
|  |  | QEN13010 | fumarate hydratase | 73 | 166 | 49.9 | 1.33E+10 |
|  |  | QEN17568 | *ibcH* | 88 | 142 | 59.8 | 9.77E+09 |
|  |  | QEN12438 | chaperonin GroEL | 90 | 137 | 56.5 | 9.08E+09 |
|  |  | QEN12190 | NAD^+^-dependent succinate- semialdehyde dehydrogenase | 83 | 110 | 47.8 | 7.96E+09 |
|  |  | QEN13004 | serine hydroxymethyl- transferase | 86 | 111 | 52 | 7.61E+09 |
|  |  | QEN12456 | aldehyde dehydrogenase | 87 | 507 | 54.8 | 6.43E+09 |

**Table S5 (continued).**

| **Band ID** | **Total proteins identified** | **Protein ID ^a^** | **Gene description** | **Coverage (%)** | **# PSMs ^b^** | **MW (kDa)** | **Abundances (Normalized)** |
| --- | --- | --- | --- | --- | --- | --- | --- |
| ~40 kDa Band | 680 proteins identified (10 contaminants, primarily keratins) | QEN17571 | *ibcK* | 60 | 475 | 34.6 | 6.40E+10 |
|  |  | QEN15752 | *adhP* | 97 | 209 | 37.1 | 8.71E+09 |
|  |  | QEN14635 | glyceraldehyde - 3-phosphate dehydrogenase | 83 | 174 | 36 | 8.53E+09 |
|  |  | QEN17565 | *ibcE* | 69 | 107 | 38.3 | 6.02E+09 |
|  |  | QEN12982 | acetyl-CoA C- acetyltransferase | 91 | 151 | 42.5 | 1.80E+09 |
|  |  | QEN15420 | homogentisate 1,2-dioxygenase | 66 | 60 | 42 | 1.04E+09 |
|  |  | QEN17568 | *ibcH* | 72 | 74 | 59.8 | 9.61E+08 |
|  |  | QEN15708 | GuaB3 family IMP dehydrogenase | 75 | 48 | 39.4 | 8.14E+08 |
|  |  | QEN14726 | acyl-CoA dehydrogenase | 59 | 70 | 44 | 7.64E+08 |
|  |  | QEN13946 | branched-chain amino acid ABC transporter substrate-binding protein | 81 | 48 | 41.8 | 6.38E+08 |

^a^ Green highlights represent plasmid-borne proteins. Blue highlights represent key chromosomal proteins discussed in the text. The table does not include identified contaminants.

^b^ PSM (peptide spectrum matches). Proteins were identified in slices from SDS-PAGE/NIR gels using LC-MS/MS analysis used a Thermo UltiMate 3000-Exploris 480 (as described in Materials and Methods).

**Fig. S5. Time course of ELW1 growth on DEE with or without IB supplementation.** The Figure shows the effect of supplementing cultures with IB during growth of ELW1 on DEE. Batch cultures of strain ELW1 were grown in glass serum bottles (700 mL) with DEE (0.5 mmol), as described in Material and Methods section of the main text. The bottles were inoculated to an initial OD_600_ of ~0.35 using washed cells previously grown in batch culture on fructose (10 mM). After approximately 72 h, IB (≤1% v/v gas phase) was added by sterile syringe into half of the cultures (⭘), whereas no IB was added to the others (⬤). The dotted red line indicates the time of IB supplementation. Culture density (OD_600_) was measured at the indicated time points. Data represents the mean and SD of three biological replicates.

**Table S6. The effects of co-substrate supplementation on DEE catabolism by ELW1.**

| **Substrate/s ^a^** | | **Substrate (+) DEE ^b^** | | | **Substrate (−)DEE** | | |
| --- | --- | --- | --- | --- | --- | --- | --- |
|  |  | **Culture density (OD_600_) ^c^** | | | **Culture density (OD_600_) ^c^** | | |
|  |  | **4 d** | **6 d** | **9 d** | **4 d** | **6 d** | **9 d** |
| Alkenes | 1-butene | 0.02 | 0.02 | 0.02 | 0.03 | 0.03 | 0.00 |
|  | 2-butene | 0.00 | 0.00 | 0.03 | 0.00 | 0.02 | 0.02 |
|  | *cis*-2-butene | 0.04 | 0.00 | 0.01 | 0.02 | 0.01 | 0.03 |
|  | *trans*-2-butene | 0.01 | 0.01 | 0.03 | 0.01 | 0.01 | 0.02 |
|  | 3-methyl-1-butene | **0.06** | **0.05** | **0.34** | 0.03 | 0.02 | 0.04 |
|  | 2-methyl-2-butene | 0.01 | 0.02 | 0.03 | 0.01 | 0.00 | 0.00 |
|  | isoprene | 0.01 | 0.01 | 0.02 | 0.02 | 0.01 | 0.03 |
|  | propene | 0.00 | 0.02 | 0.01 | 0.01 | 0.03 | 0.01 |
|  | ethylene | 0.02 | 0.05 | 0.07 | 0.02 | 0.02 | 0.03 |
| Alkanes | methane | 0.00 | 0.05 | 0.08 | 0.01 | 0.02 | 0.02 |
|  | propane | 0.02 | 0.04 | 0.06 | 0.00 | 0.02 | 0.01 |
|  | isobutane | 0.02 | 0.06 | 0.04 | 0.04 | 0.01 | 0.01 |
| Alkynes | propyne | 0.05 | 0.02 | 0.03 | 0.04 | 0.01 | 0.03 |
|  | IB + propyne | 0.01 | 0.00 | 0.01 | 0.02 | 0.00 | 0.00 |
| Ibc pathway metabolites | IB | **0.19** | **0.59** | **1.19** | 0.08 | 0.06 | 0.05 |
|  | IBO | 0.1 | 0.11 | 0.22 | 0.1 | 0.12 | 0.15 |
|  | MPD | 0.12 | 0.13 | 0.15 | 0.08 | 0.11 | 0.11 |
|  | 2-HIBA | **0.03** | **0.08** | **0.30** | 0.02 | 0.03 | 0.03 |

**Table S6 (continued).**

| **Substrate/s ^a^** | | **Substrate (+) DEE ^b^** | | | **Substrate (−)DEE** | | |
| --- | --- | --- | --- | --- | --- | --- | --- |
|  |  | **Culture density (OD_600_) ^c^** | | | **Culture density (OD_600_) ^c^** | | |
|  |  | **4 d** | **6 d** | **9 d** | **4 d** | **6 d** | **9 d** |
| Control | DEE only | 0.01 | 0.02 | 0.02 |  |  |  |
|  | Fructose | 0.26 | 0.31 | 0.34 | 0.24 | 0.24 | 0.20 |

^a^ Gaseous substrates (∼0.75%, v/v, gas phase) and (~37.5 µmol) liquid substrates added as aqueous or neat solutions to a final concentration of ~1.5 mM.

^b^ 0.25 mmol DEE was added as a neat solution (final concentration of ~9.5 mM) to designated bottles.

^c^ All Initial culture OD_600_ ≤ 0.01. Data represents the mean of two biological replicates.

Red text = Notable differences in growth.

Batch cultures of ELW1 were grown in glass serum bottles (160 mL) as described in the Materials and Methods section of the main text. The bottles were inoculated with ELW1 cells to an initial OD_600_ of ~0.01. The bottles were sealed and then gaseous substrates (∼0.75%, v/v, gas phase) or liquid substrates (~37.5 µmol) were added. DEE (0.25 mmol) was added by syringe to designated bottles. Culture density (OD_600_) was measured at the indicated times.

**Table S7. Consumption of ethanol and 2-methyl-1,2-propanediol by resting IB-grown and DEE-grown cells of ELW1.**

|  | **ethanol ^a^** | | **2-methyl-1,2-propanediol ^a^** | |
| --- | --- | --- | --- | --- |
|  | **(−) 16HD** | **(+) 16HD** | **(−) 16HD** | **(+) 16HD** |
| **IB-grown cells ^b^** | 8.1 ±1.1 | 0.6 ±0.6 | 124.0 ±5.1 | 118.5 ±2.2 |
| **DEE-grown cells ^b^** | 92.0 ±6.4 | 78.7 ±2.8 | 86.7 ±3.7 | 76.9 ±15.5 |

^a^ An initial amount of ~2.5 µmol of ethanol or 2-methyl-1,2-propanediol (MPD) was added from 0.1 M aqueous stocks. Ethanol consumption was measured after 40 min. MPD consumption was measured after 60 min.

^b^ Data represents the whole-cell specific activities in nmol.min^-1^.mg protein^-1^. Data represents the mean and SD from two biological replicates. 1,6-heptadiyne (16HD) was used to measure the potential inactivation of alcohol dehydrogenases by a diyne probe.

Batch cultures of ELW1 were grown on IB (~10%, v/v, gas phase) or DEE (5 mM) to late log phase, washed, and resuspended in PB, as described for resting cell assays in the Materials and Methods section of the main text. Resting cell suspensions (1 mL) were then probed with 0.5 µmol 1,6-heptadiyne (16HD), or an equivalent volume of DMSO, for 20 min prior to the addition of ethanol or MPD. Ethanol or MPD were then added to each reaction vial to a final concentration of ~2.5 mM. Substrate consumption was measured by gas chromatography, as described for liquid-phase analytes in Materials and Methods section of the main text.

**References**

Altschul SF, Madden TL, Schäffer AA, et al (1997) Gapped BLAST and PSI-BLAST: a new generation of protein database search programs. Nucleic Acids Res 25:3389–3402. https://doi.org/10.1093/nar/25.17.3389

Camacho C, Coulouris G, Avagyan V, et al (2009) BLAST+: architecture and applications. BMC Bioinformatics 10:421. https://doi.org/10.1186/1471-2105-10-421

Jones DT, Taylor WR, Thornton JM (1992) The rapid generation of mutation data matrices from protein sequences. Comput Appl Biosci CABIOS 8:275–282. https://doi.org/10.1093/bioinformatics/8.3.275

Ko JH, Jeong KH, Son SB, Lee JY (2025) Structural analysis of ExaC, an NAD^+^-dependent aldehyde dehydrogenase, from *Pseudomonas aeruginosa*. Biochem Biophys Res Commun 742:151077. https://doi.org/10.1016/j.bbrc.2024.151077

Kumar S, Stecher G, Suleski M, et al (2024) MEGA12: Molecular Evolutionary Genetic Analysis Version 12 for Adaptive and Green Computing. Mol Biol Evol 41:msae263. https://doi.org/10.1093/molbev/msae263

Thomas LM, Harper AR, Miner WA, et al (2013) Structure of *Escherichia coli* AdhP (ethanol-inducible dehydrogenase) with bound NAD. Acta Crystallograph Sect F Struct Biol Cryst Commun 69:730–732. https://doi.org/10.1107/S1744309113015170
